# Recent advances in the utilization of covalent organic frameworks (COFs) as electrode materials for supercapacitors

**DOI:** 10.1039/d3sc04571d

**Published:** 2023-11-07

**Authors:** Shen Xu, Jinghang Wu, Xiang Wang, Qichun Zhang

**Affiliations:** a Department of Materials Science and Engineering, City University of Hong Kong Hong Kong SAR 999077 P. R. China qiczhang@cityu.edu.hk; b Department of Chemistry, Center of Super-Diamond and Advanced Films (COSDAF), City University of Hong Kong Hong Kong SAR 999077 P. R. China

## Abstract

Due to their excellent stability, ease of modification, high specific surface area, and tunable redox potentials, covalent organic frameworks (COFs) as potential electrodes in supercapacitors (SCs) have raised much research interest because these materials can enable the achievement of high electric double-layer supercapacitance and high pseudocapacitance. Here, the design strategies and SC applications of COF-based electrode materials are summarized. The detailed principles are introduced first, followed by discussions on strategies with diverse examples. The updated advances in design and applications are also discussed. Finally, in the outlook section, we provide some guidelines on the rational design of COF-based electrode materials for high-performance SCs, which we hope will inspire novel concepts for COF-based supercapacitors.

## Abstract 2

This review summarizes the recent advances in the electrode application of covalent organic frameworks (COFs) for supercapacitors, including the design strategies from the molecular scale to morphology control level and their device performance.

## Graphical abstract

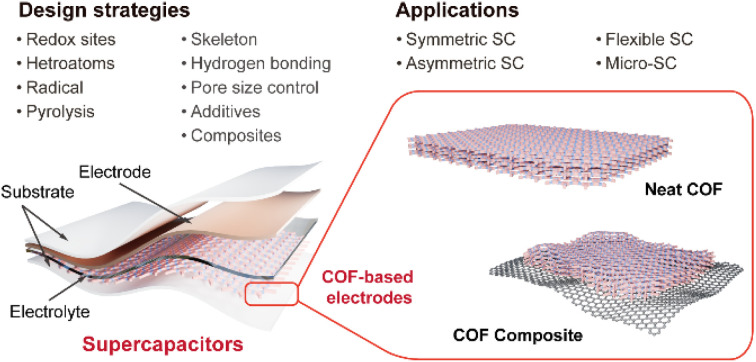

## 1. Introduction

Although current power generation is dominated by traditional fossil energy,^1^ the exhausting fossil sources and related environmental issues are forcing people to find alternatives.^2^ A rising share of clean and renewable energy in the total energy generation has been witnessed in recent years. For instance, wind and solar energy contributed to 10.5% of energy generation in 2021, compared to only 0.7% in 2012.^3^ However, the increasing production of renewable energy and its discontinuous generation nature, as well as the usage of electric vehicles and portable electronic devices have led to the rapidly growing demand for electrochemical energy storage (EES) devices. From 2022 to 2030, an estimated 387 GW/1143 GW h of new energy storage capacity will be added globally.^4^

Rechargeable batteries and capacitors are two representative types of EES devices.^5–10^ Rechargeable batteries, based on redox reactions, have been widely applied in a variety of fields for decades due to their high capacity and energy density. On the other hand, capacitors exhibit ultrahigh power density, outstanding cycling stability, and ultrafast charging/discharging rate owing to the utilization of electrochemical double-layer capacitance (EDLC).^11^ Especially, supercapacitors (SCs) have been widely considered as a partial combination of batteries and traditional capacitors due to the integration of their advantages.^12,13^ The capability of SCs to combine EDLC and pseudocapacitance (faradaic redox reactions) together to acquire balanced high energy density and power density makes them more promising for various applications.^14^ The physical adsorption mechanism of traditional EDLCs enables surface-controlled fast adsorption and desorption for high power density and short charging/discharging time.^15^ In contrast, the principle of pseudocapacitance (PC) usually involves surface-controlled faradaic redox reactions at the interface of the electrolyte and the electrode (sometimes mixed with diffusion-controlled faradaic reactions within the bulk electrode).^16^ The difference in the faradaic reaction mechanism endows PCs with much higher power density than batteries. Traditional materials such as electrodes in SCs are mainly classified into two types: carbon materials and layered materials utilizing EDLC; as well as hetero-atom doped carbons, transition metal oxides/sulfides, and organic redox-active materials based on the pseudocapacitive mechanism.^17–19^ However, the increasingly complicated application scenarios have raised the widespread demand for high performance electrode materials, which are the key element for high performance SCs.

As one class of star materials, covalent organic frameworks (COFs) have attracted many scientists' attention. COFs are one type of crystalline porous polymeric material.^20,21^ Since the first report by Yaghi^22^ in 2005, their highly ordered and designable structure, large specific surface area (SSA), and tunable photoelectronic properties enable their wide applications in catalysts, photoelectric devices, sensing, energy conversion, and energy storage.^23–27^ The ordered crystalline structure (especially for two-dimensional COFs) and large SSA are favorable for efficient EDLC, while the capability of introducing redox active sites on the COF skeleton is beneficial to PC.^28^ Therefore, COFs can be employed as a promising platform for the combination of EDLC and PC in a single system. Dichtel and colleagues for the first time realized the great potential of COFs and employed DAAQ-TFP COF as the electrode material for SCs.^29^ Since then, COF-based electrode materials in SCs have attracted tremendous attention not only to dig the potential of COFs themselves, but also to combine them with other materials that complement them.^30^ Furthermore, the free-standing and flexible electrodes realized by COF-based composites enable diverse devices including flexible and transparent SCs.^31,32^

Different from battery applications, the contributions of COF-based electrodes for SCs have rarely been summarized and discussed. Nevertheless, some excellent reviews on the related topics have been published in recent years, covering the fundamental requirements and design principles, theoretical insight into charge and mass transport, and historical introduction.^33–38^ Considering the increasing concerns and rapid development of COF-based SCs, a review with updated progress in materials design and device application is highly desirable. In this contribution, the design strategies from the molecular scale to morphology control level will be discussed firstly. Then the device performance of COF-based electrodes will be summarized. The achievements and challenges in this field will be presented at last. The representative works that can provide a valuable design concept of COFs and COF-based composites, as well as the most recent progress on both materials and devices will be mainly focused on. Hopefully, this review will provide some guidelines in the design of novel COF-based SCs.

## 2. Electrochemical characterization and mechanisms of COF-based SCs

To evaluate the supercapacitive performance of COF-based electrodes, a series of indexes are introduced including specific capacitance, cycling stability, rate capability, energy density, and power density. These parameters can comprehensively describe the upper limit and practical potential of the electrode materials, which are presented in this section. Meanwhile, the representative electric storage mechanisms are also introduced in this section.

### 2.1 Electrochemical parameters

In this part, the mostly used electrochemical indicators and parameters will be introduced. The relationship between these indicators and design strategies will be discussed in detail in Section 3.

#### 2.1.1 Specific capacitance

Specific capacitance (*C*_sp_) describes the capability of charge storage of SCs. According to the different quantitative measurement methods of electrode materials, the *C*_sp_s can be divided into gravimetric capacitance, areal capacitance, and volumetric capacitance with F g^−1^, F cm^−2^, and F cm^−3^ as the units, respectively. Gravimetric capacitance is widely used in half-cells to estimate the charge storage capability of electrode materials, while areal capacitance and volumetric capacitance are commonly used in full-cells for the overall assessment of the devices. Benefitting from the combination of PC and EDLC and their integrated advantages, COF-based SCs generally exhibit better capacitive performance. For COF-based electrodes, the total capacitance can be affected by the number of redox sites, specific surface area, and pore size. For SC devices, the electrolyte system and solid–electrolyte interface (SEI) also play important roles in *C*_sp_.

#### 2.1.2 Rate capability

Rate capability is usually expressed as the specific capacitance retention percentage at high scan rate/current density compared to that at low scan rate/current density. Apart from the above influence factors, *C*_sp_ is also dependent on the test conditions. Generally, charge storage processes can be fully finished at a low scan rate/current density, which results in a high *C*_sp_. For the EDLC, the capacitance only decreases a little bit owing to the ultrahigh rate of ion insertion and extraction processes, representing good rate capability. For the PC, the non-faradaic redox reactions also have high reaction rate, which is comparable to EDLC. However, the bulk electrode material could hinder the access of ions to the redox sites at high scan rate/current density. Besides, the unavoidable faradaic-type redox reaction with relatively low rate in some COF-based electrodes and poor conductivity can also lead to poor rate capability.

#### 2.1.3 Cycling stability

As another important indicator of SCs to evaluate the available working lifespan, cycling stability is generally indicated as the specific capacitance retention after a certain number of charging/discharging cycles. On the electrode level, the physical and chemical stability of electrode materials, conductivity, resistive losses from sluggish electron and ion transport, and compatibility of the electrode with electrolyte play crucial roles.

#### 2.1.4 Energy and power density

Energy density is defined as the stored energy per unit (mass, area, or volume), while power density represents the output power per unit. Higher energy density indicates more energy can be stored in SCs of the same size, while high power density demonstrates a high charging/discharging rate. Similar to the specific capacitance, the unit of energy/power density can also be divided into W h kg^−1^/W kg^−1^, W h cm^−2^/W cm^−2^, and W h m^−3^/W m^−3^, respectively. It is well known that batteries possess ultrahigh energy density but low power density. In contrast, SCs, especially EDLCs, have high power density and relatively low energy density. The difference between them is mainly due to the different charge storage kinetics. The diffusion-controlled faradaic redox reaction of batteries can acquire high capacity, but the low reaction rate leads to unsatisfactory rate capability and low power density. Conversely, as illustrated above, the ultrahigh rate of ion insertion and extraction processes of traditional EDLCs leads to ultrahigh power density, but the limited specific capacitance results in low energy density. With the combination of EDLCs and PCs, COF-based SCs have great potential to obtain high energy density, while maintaining the high power density of EDLCs. Notably, the electrolyte system also impacts on the energy/power density which will be discussed later.

### 2.2 Storage mechanisms of COF-based electrodes

**Scheme 1:**
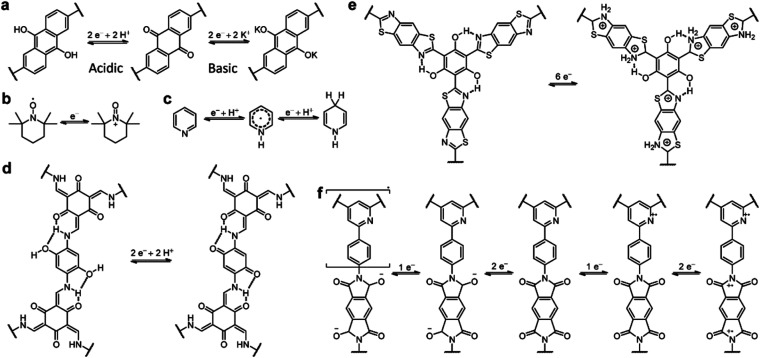
The representative pseudocapacitive charge storage mechanisms of COF-based SCs.

Owing to the coexistence of PC and EDLC, the storage mechanism of COF-based SCs is quite complicated. The redox-active sites on the COF skeleton impart the redox reaction-based PC, while the porous structure with high SSA enables the realization of EDLC. As shown in Scheme 1, the mechanism of PC is similar to that of batteries. It is worth noting that, although the ideal mechanism of PC is based on the non-faradaic reaction, the mechanism of most reported COF-based SCs involves both the faradaic and non-faradaic redox reactions. Therefore, the rate capability and power density of these devices are not as good as for EDLCs, which is one of the main obstacles to be solved. Apart from the redox mechanisms for PC, the EDLC mechanisms of COF-based electrodes are seldom discussed. The limited cases will be introduced in the Strategy section.

## 3. Design strategies of COF-based electrode materials

**Fig. 1:**
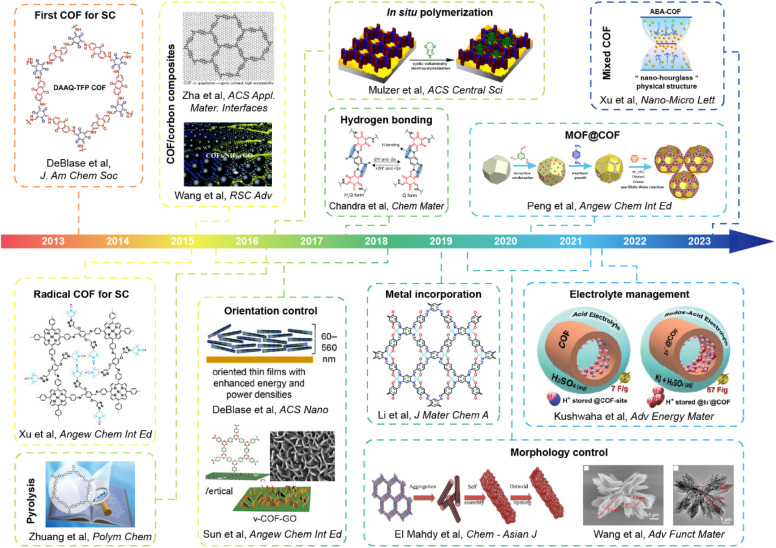
The development of the design strategy of COF-based materials for SCs.

Given the advantages of low density, abundant functionalizing sites, large specific surface area, porous structure, and outstanding stability, COFs (especially 2D COFs) are considered as a series of excellent candidates of electrode materials for SCs. Since the first attempt at utilizing COFs as the electrodes of SCs ten years ago, many efforts have been made seeking the improvement of the capacitance, stability, and energy density of SCs. The main obstacles that hinder the efficiency improvement of COF-based SCs are the intrinsic poor conductivity of most COFs and the unsatisfactory accessibility to the redox sites. Based on these considerations, most works focus on introducing different redox sites, manipulating pore size, managing morphology on the molecular level, or forming composites with polymers and conductive carbons. Sometimes, several methods were integrated to realize the synergistic enhancement. In this section, design strategies of COF-based electrode materials for SCs that can realize the improvement of specific capacity, energy density, stability, or other performance parameters will be discussed (Fig. 1). We aim to provide some guidelines on the materials design, and the detailed device performance will be discussed in the next section.

### 3.1 General design principles

**Scheme 2:**
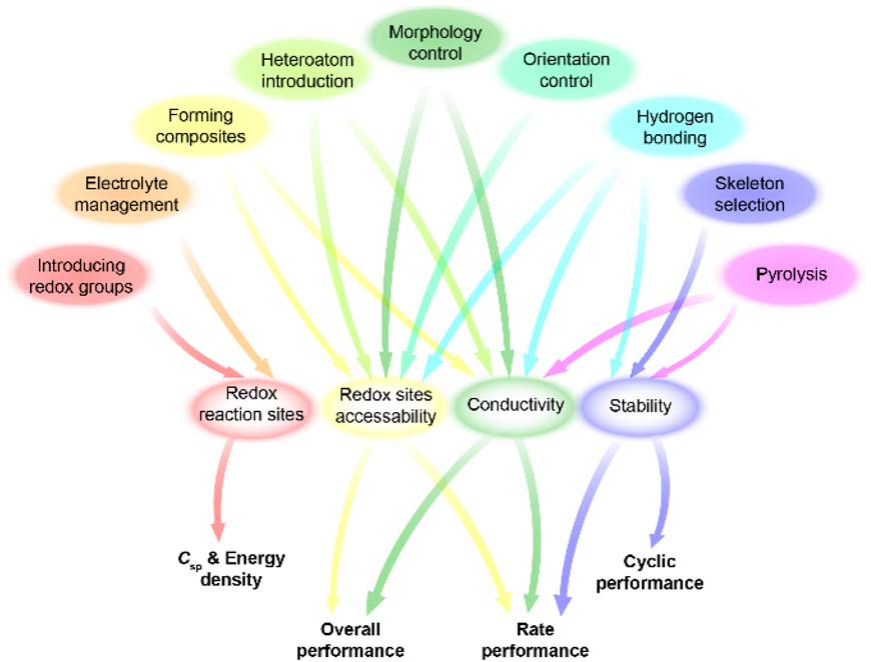
Relationship between strategies and device performance.

Different design strategies lead to diverse performance of SCs (Scheme 2). The original concept of COF-based materials for supercapacitor applications is to utilize both the PC and EDLC. Therefore, the introduction of redox groups can effectively increase the pseudocapacitance, thus increasing the total specific capacitance (*C*_sp_). On the basis of fixed voltage, the energy density of the SC can also be improved. Similarly, employing redox active electrolyte is favorable for the increase of *C*_sp_ and energy density.

To acquire high crystallinity, traditional COFs are usually synthesized through reversible condensation reactions. Therefore, the linkage of COFs cannot resist harsh conditions such as strong acidic and basic electrolyte for SCs. Thanks to the growth of reticular chemistry and materials science, many new kinds of COFs with robust linkages have been developed. The improved chemical and thermal stability of the electrodes based on robust COFs are beneficial to rate performance and long-term cycling stability at high current density. Pyrolysis of COFs can also provide improved stability. The high temperature treatment of COFs generates heteroatom-doped carbon materials with a mixture of conjugated and nonconjugated backbone.

The first COF-based SC reveals the great potential of COFs in enhancing capacitance and energy density on the one hand, but on the other hand, it exposes some obstacles of bulk COFs including low accessibility of redox sites and low intrinsic conductivity. To solve these problems, some strategies were proposed. For the neat COFs, introducing heteroatoms such as sulfur and metal atoms or hydrogen bonds can effectively increase the conductivity, thus realizing high charge transfer and mass transport for improved redox site accessibility. Besides, controlling the orientation of COF film can achieve an improved charge transport channel for better accessibility.

COFs are also able to form composites with conductive materials to improve the conductivity. For instance, a huge improvement in conductivity can be achieved when growing COFs on graphene or carbon nanotubes (CNTs). Moreover, the thickness of the COF film can be well controlled under optimal conditions for high accessibility of redox sites. Incorporating conductive polymers in the pore of COFs is able to realize similar effects. Besides, the morphology of neat COFs or COF composites can be tuned to acquire an extra enhancement in accessing redox sites and conductivity. The special COF morphologies such as vertically oriented nanosheets or inside-out Ostwald ripening-driven hollow structure provide higher SSA and redox site accessibility. The hierarchical morphologies of composites ensure unimpeded charge and mass transport in the electrode. Reasonably, the simultaneous utilization of several strategies can realize a synergistic enhancement of total supercapacitive performance. Detailed strategies will be discussed in the following parts.

### 3.2 Introducing redox-active groups

Introducing redox sites onto the COF skeleton is the most effective way to increase the pseudocapacitance. Similar to organic electrode materials for batteries, heteroatom-containing groups including carbonyl, pyridine, triazine, and pyrrole can be introduced for the faradaic reactions. However, the different energy storage mechanisms for batteries and pseudocapacitors result in the different effectiveness of redox groups in performance improvement.

#### 3.2.1 Carbonyl groups

**Fig. 2:**
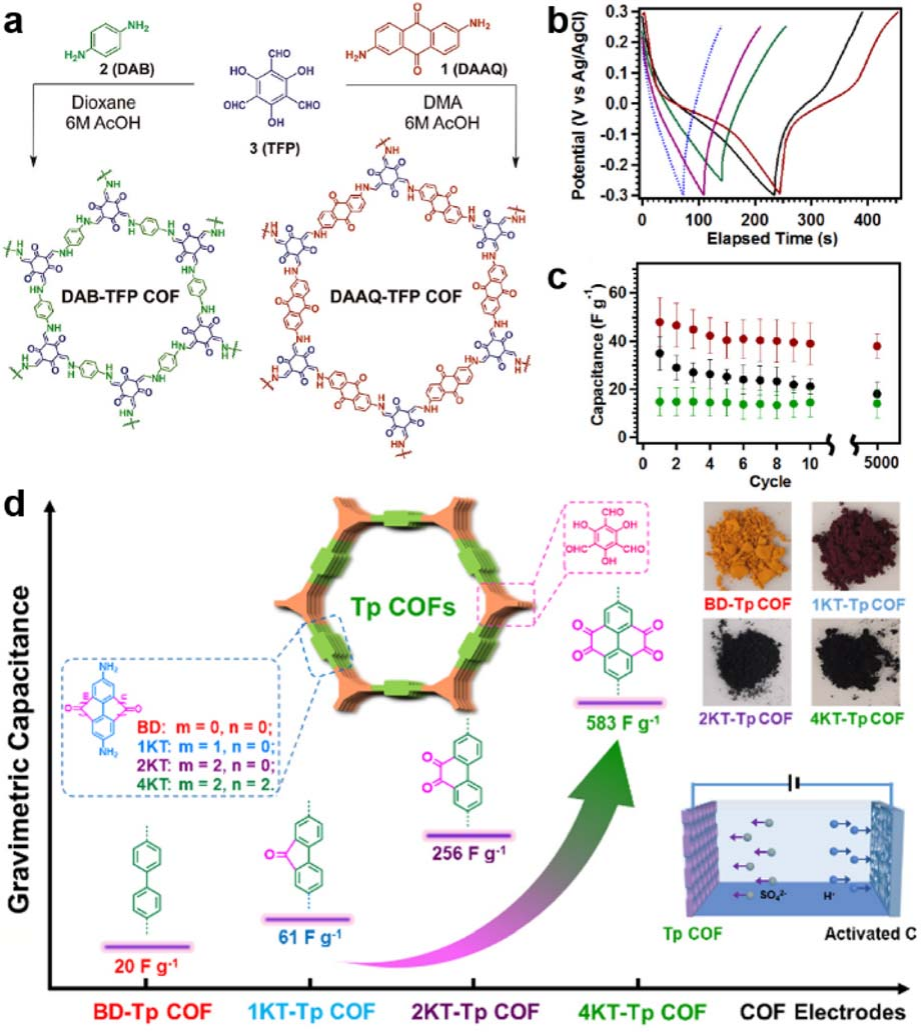
(a) Synthetic routes and structures of DAAQ-TFP COF and DAB-TFP-COF. (b) GCD response and (c) average discharge capacitances at 0.1 A g^−1^ after different cycle numbers of DAAQ-TFP COF, DAAQ monomer, and DAB-TFP COF. Reprinted from ref. 29. Copyright 2013, American Chemical Society. (d) Schematic illustration, specific capacitance, and photos of Tp-COFs with different redox groups. Reprinted from ref. 41. Copyright 2020, Chinese Chemical Society.

Carbonyl groups have been widely used in constructing redox-active molecules for organic electrodes.^39,40^ Therefore, it is reasonable to introduce carbonyl-containing moieties in COFs to increase the capacitance. In 2013, the Dichtel group for the first time developed β-ketoenamine-linked 2D COFs as an electrode material for SCs (Fig. 2a).^29^ Meanwhile, the anthraquinone unit was introduced as the active site to increase the pseudocapacitive contribution (DAAQ-TFP COF). Compared with the counterpart without the anthraquinone unit (DAB-TFP COF), DAAQ-TFP COF shows several-fold higher *C*_sp_. It is worth noting that the nearly triangular galvanostatic charge–discharge (GCD) curves of DAB-TFP COF demonstrate the lower redox activity of β-ketoenamine than anthraquinone (Fig. 2b and c).

To maximize the redox contribution to total capacitance, Li and colleagues introduced an orthoquinone group into the COF skeleton (Fig. 2d).^41^ Including the reference compounds, four COFs (BD-Tp COF, 1KT-Tp COF, 2KT-Tp COF, and 4KT-Tp COF) exhibit apparently increased *C*_sp_ from 20 to 61, 256, and 583 F g^−1^ at 0.2 A g^−1^, with an increasing number of ketones (from 0 to 1, 2, and 4) within the skeleton, respectively. Reasonably, the pseudocapacitive contribution for 2KT-Tp COF and 4KT-Tp COF reach as high as 63% and 82%. Apart from the increased redox sites, the extended conjugation upon reduction, simultaneous participation of the adjacent carbonyl groups, and enhanced charge transfer should play more important roles in the capacitance improvement.

#### 3.2.2 Nitrogen-containing groups

**Fig. 3:**
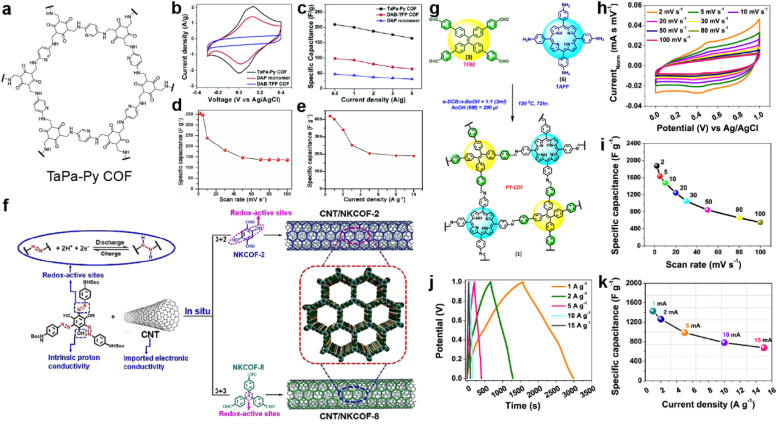
(a) Structure of TaPa-Py COF. (b) CV curves and (c) discharge capacitances at different current densities of TaPa-Py COF, DAP monomer, and DAB-TFP COF. Reprinted from ref. 42. Copyright 2016, Royal Society of Chemistry. Specific capacitances of TaPa-Py COF (d) at different scan rates and (e) current densities. Reprinted from ref. 43. Copyright 2017, Wiley-VCH GmbH. (f) The fabrication of CNT/NKCOFs. Reprinted from ref. 44. Copyright 2021, Wiley-VCH GmbH. (g) Schematic diagram of the synthetic route to PT-COF. (h) Cyclic voltammograms and (i) *C*_sp_ of PT-COF at different scan rates. (j) GCD plot and (k) *C*_sp_ of PT-COF at different current densities. Reprinted from ref. 45. Copyright 2021, American Chemical Society.

Nitrogen-containing groups including pyridine, phenazine, and triazine are also known as effective redox-active sites. In 2016, Khattak *et al.* prepared a β-ketoenamine-linked COF *via* the condensation reactions between diaminopyridine (DAP) and triformylphloroglucinol (TFP) (Fig. 3a).^42^ The obtained TaPa-Py COF exhibits a combination of PC and EDLC according to cyclic voltammetry (CV) curves, while the reference COF, constructed with diaminobenzene (DAB) and TFP, only shows EDLC (Fig. 3b). Besides, the Brunauer–Emmett–Teller (BET) SSA of TaPa-Py COF (687 m^2^ g^−1^) is much larger than that of DAB-TFP COF (385 m^2^ g^−1^). As a result, the electrode based on TaPa-Py COF shows a more than doubled *C*_sp_ of 180.5 F g^−1^ at 20 mV s^−1^ in a three-electrode system calculated by CV. Moreover, TaPa-Py COF manifests 4 times higher *C*_sp_ than DAB-TFP COF at the higher scan rate, illustrating the better rate capability of TaPa-Py COF. The GCD result and two-electrode system show similar results (Fig. 3c–e).

Bhanja and co-workers first employed a triazine-based COF as an electrode material under acidic conditions.^43^ Both the triazine and keto units from 2,6-diformyl-4-methylphenol (DFP) can act as the redox sites for the PC. Combined with a high BET SSA of 651 m^2^ g^−1^ and coexistence of micro- and mesopores, high specific capacities of 354 and 418 F g^−1^ at 2 mV s^−1^ and 0.5 A g^−1^ were obtained from CV and GCD tests in a three-electrode system, respectively, which are comparable to those of carbon-based electrode materials. However, a sharp decrease is observed when increasing the scan rate or current density, illustrating the poor rate capability mainly due to the low intrinsic conductivity.

Installing azo groups into the COF skeleton is also favorable for improving the PC. Moreover, the hydrophilic properties of the azo group could effectively optimize the wettability of the azo-COF-based electrode in aqueous electrolyte. By solvothermal condensation reactions between the Azo-NHBoc monomer and phenyl amine compounds, two azo-containing imine-COFs (NKCOF-2 and NKCOF-8) were synthesized by Yang *et al.* (Fig. 3f).^44^ With the assistance of CNT to upgrade the conductivity, up to 440 F g^−1^ at 0.5 A g^−1^ can be achieved with rate retention of 49% (216 F g^−1^) at 20 A g^−1^ and cyclic retention of 91% after 10 000 cycles at 10 A g^−1^.

Porphyrin is well known as a nitrogen-rich semiconducting molecule. Thus, Patra and Bhattacharya constructed an imine-linked PT-COF through the condensation reaction of TAPP (5,10,15,20-tetrakis(*para*-amino phenyl)-porphyrin) with TFBE (1,1,2,2-tetrakis(4-formyl-(1,1′-biphenyl))-ethane) (Fig. 3g).^45^ The as-prepared COF possesses excellent crystallinity, large BET SSA of 1998 m^2^ g^−1^, and moderate pore size of 1.45 nm, which favor the application in SCs. Excitingly, according to the CV test in Fig. 3h and i, the electrode using bulk COF without conductive carbon shows very high *C*_sp_ of 1875 F g^−1^ at a scan rate of 2 mV s^−1^, which is among the best results for COF-based electrodes. Capacitance of more than 1200 and 550 F g^−1^ can be retained at higher scan rates of 20 and 100 mV s^−1^. As shown in Fig. 3j and k, the GCD test also delivers high *C*_sp_ of 1443 F g^−1^ at a current density of 1 A g^−1^. However, the loaded mass of the COF is as low as 20 μg. Integrating with the moderate rate performance, the practical utilization of this COF is limited. This should be attributed to the relatively low conductivity of the bulk COF of 7.06 × 10^−8^ S cm^−1^.

#### 3.2.3 Radical groups

**Fig. 4:**
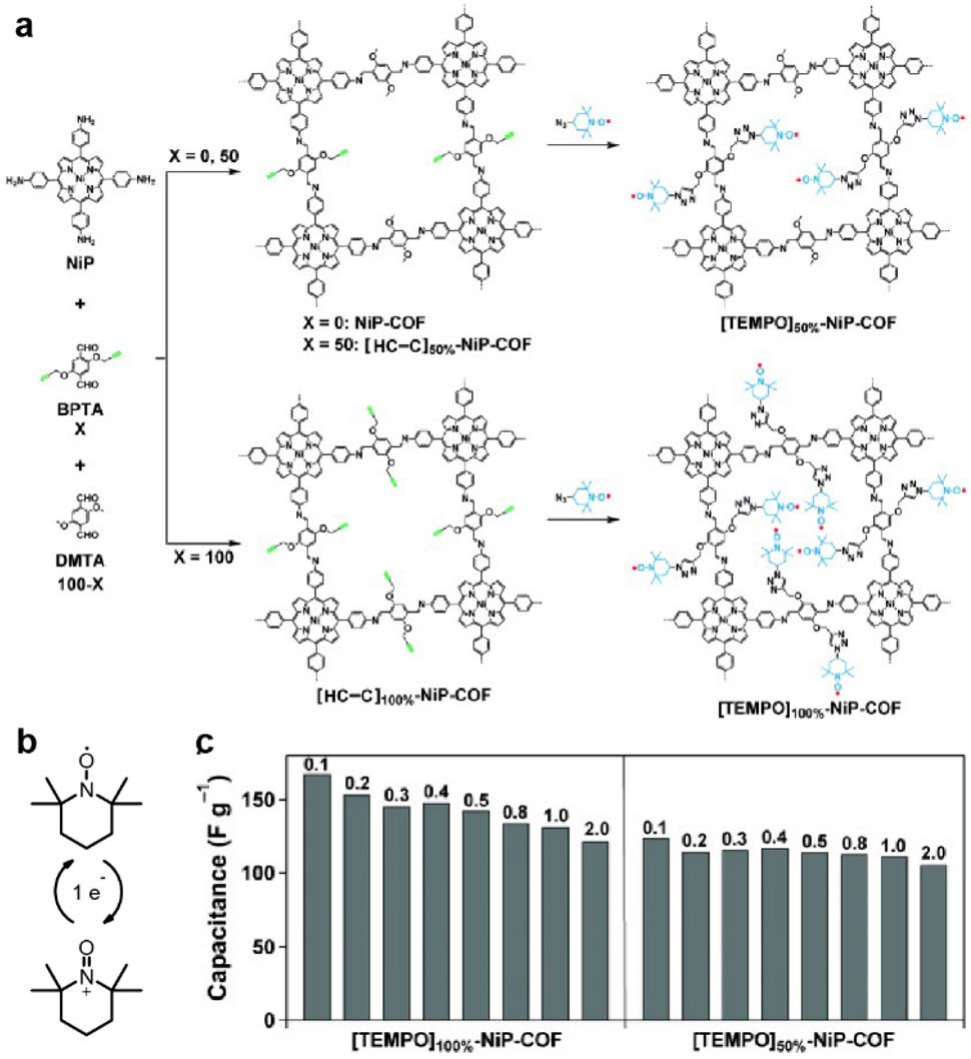
(a) Synthesis of radical COFs ([TEMPO]100%-NiP-COF and [TEMPO]50%-NiP-COF) with TEMPO radicals immobilized on the walls by functionalization of [HC≡C]100%-NiP-COF and [HC≡C]50%-NiP-COF. (b) The charge–discharge process of TEMPO based on a one-electron redox reaction. (c) Capacitance of [TEMPO]100%-NiP-COF and [TEMPO]50%-NiP-COF at different current densities. Reprinted from ref. 46. Copyright 2015, Wiley-VCH Verlag GmbH.

The first radical COF was synthesized by Xu *et al.* in 2015 (Fig. 4a).^46^ The classic radical moiety TEMPO (2,2,6,6-tetramethylpiperidine-1-oxyl) was grafted onto the Ni-porphyrin-based imine COF by click reaction. The reversible switch between the oxoammonium cation and the neutral radical of TEMPO makes it redox active (Fig. 4b). Although the BET SSA decreases significantly after grafting the TEMPO units (264 and 5.2 m^2^ g^−1^ for [TEMPO]50%-NiP-COF and [TEMPO]100%-NiP-COF, respectively), specific capacitances of 167 and 124 F g^−1^ at 0.1 A g^−1^ were obtained in a three-electrode system using neutral electrolyte, illustrating the feasibility of employing radical COFs as the electrode materials for SCs (Fig. 4c).

### 3.3 Forming composites

The intrinsic low conductivity of most COFs is the main hindrance that restricts the performance of COF-based SCs. To solve this problem, COFs could be composited with other conductive materials including conductive polymers, carbon materials, and MOFs.^47^

#### 3.3.1 Compositing with carbon materials

Carbon materials are well established as conducting materials and widely applied in SCs. Combining COFs with carbon materials through either simple mixing or *in situ* condensation can acquire the integration of both advantages.

**Fig. 5:**
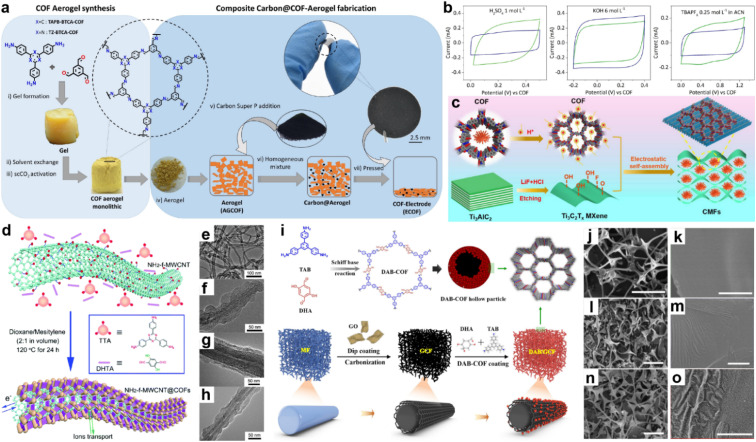
(a) Fabrication of COF electrodes (ECOFs). (b) Cyclic voltammetry at 100 mV s^−1^ for TAPB-BTCA-ECOF (blue) and TZ-BTCA-ECOF (green) using electrolytes of 1 mol L^−1^ H_2_SO_4_, 6 mol L^−1^ KOH, and 0.25 mol L^−1^ TBAPF_6_ in CAN (left to right). Reprinted from ref. 48. Copyright 2022, Wiley-VCH GmbH. (c) Schematic illustration of CMF preparation. Reprinted from ref. 52. Copyright 2023, Elsevier. (d) Schematic illustration of COF_TTA–DHTA_ shells on the NH_2_-f-MWCNT surface. COF_TTA–DHTA_ was constructed from TTA and DHTA *via* imine linkages. TEM images of (e) NH_2_-f-MWCNT, and NH_2_-f-MWCNT@COF_TTA–DHTA_ synthesized at (f) 5 mg mL^−1^ and (g) 10 mg mL^−1^ NH_2_-f-MWCNT at 120 °C for 24 h, and (h) MWCNT@COF_TTA–DHTA_ (10 mg mL^−1^ MWCNT). Reprinted from ref. 50. Copyright 2017, Royal Society of Chemistry. (i) Schematic illustration of the synthesis route for DAB/GCF. SEM images of cMF (j and k), GCF (l and m), and DAB/GCF (n and o). Scale bar: (j, l and n) 50 μm, (k, m and o) 1 μm. Reprinted from ref. 53. Copyright 2022, Elsevier.

Martín Illán *et al.* simply mixed two imine-COF aerogels with 30% Super P and then pressed them under 120 MPa to get free-standing electrodes (Fig. 5a).^48^ Nevertheless, moderate crystallinity and BET SSA of 470 and 565 m^2^ g^−1^ for TAPB-BTCA-ECOF and TZ-BTCA-ECOF can be retained under this ultrahigh pressure, while the conductivity increased by seven orders of magnitude compared with the corresponding COF-aerogels. As shown in Fig. 5b, the two electrodes display typical rectangle-like CV patterns in acidic and basic aqueous electrolytes and organic electrolyte, except for TZ-BTCA-ECOF, which shows nearly negligible pseudocapacitive contribution in organic media. At a scan rate of 100 mV s^−1^, areal specific capacitances of 11.2 and 8.95 mF cm^−2^ are achieved for TAPB-BTCA-ECOF and TZ-BTCA-ECOF. Notably, *C*_sp_ of 8.95 mF cm^−2^ can still be reached by TAPB-BTCA-ECOF at 1000 mV s^−1^, demonstrating outstanding EDLC rate capability of the ECOF.

In 2015, Wang and co-workers constructed a COF/rGO (reduced graphene oxide) composite *via in situ* polymerization of imine COF on NH_2_ modified rGO.^49^ The PXRD pattern of COF/NH_2_-rGO reveals the mixed signals of precursors. When employing this composite as the active electrode material in neutral electrolyte, the CV test illustrates the co-existence of EDLC and PC. Meanwhile, a high specific capacity of 533 F g^−1^ at a current density of 0.2 A g^−1^ is obtained in the GCD test, which is even higher than the sum of the values for bulk COF and NH_2_-rGO. Excitingly, 90% of the initial capacitance can be retained at a current density of 2 A g^−1^, indicating much improved rate capability.

Carbon nanotubes have been demonstrated to be one of the promising conductive carbon additives for energy storage devices. In 2017, COF_TTA–DHTA_ was *in situ* synthesized on the amino-functionalized carbon nanotubes (NH_2_-f-MWCNTs) by Sun *et al.* (Fig. 5d).^50^ The low thickness (∼20 nm) and perpendicular orientation of the COF shell enable the easy penetration of electrolyte (Fig. 5e–h). *C*_sp_ of 127.5 F g^−1^ at 0.4 A g^−1^ and good cycling stability can be achieved. Similarly, Han and co-workers combined TpPa-COF with single-walled carbon nanotubes (SWCNT).^51^*C*_sp_ of 153 F g^−1^ at the current density of 0.5 A g^−1^ was realized in acidic electrolyte. The equivalent series resistance (*R*_s_) is 4.9 Ω, indicating good conductivity of the composite. However, the rate capability is poor as only ∼35 F g^−1^ of the *C*_sp_ can be retained at the current density of 2 A g^−1^.

As a kind of transition metal carbide material, MXene has also been employed as the substrate to form composites with COFs. Recently, An *et al.* utilized DAAQ-COF and few-layered MXene to construct a CMF composite *via* a cation-driven self-assembly process.^52^ The electrostatic attraction between protonated COF and MXene with negative charge induces the rapid wrapping and embedment of the flower-like COF cluster into the twisted MXene sheet to form a 3D conductive network without over-stacking of MXene (Fig. 5c). Using the optimal mass ratio of 3 : 1 for DAAQ-COFs and Ti_3_C_2_Tx MXene, a highest *C*_sp_ of 361 F g^−1^ at 1 A g^−1^ can be obtained in a three-electrode system in acidic electrolyte, which is 15- and 1.5-fold the capacitance of DAAQ-COF and MXene, respectively. Furthermore, 81.5% of the capacitance can be retained at 10 A g^−1^, representing excellent rate capability.

Because most COFs can be deposited on the surface of carbon materials, the morphology of the composite is mainly determined by the carbon materials, which is of importance to the final capacitive performance. Recently, Dong and co-workers employed a melamine foam/GO carbonized 3D framework (GCF) as the platform to grow 2D DAB-COF (Fig. 5i).^53^ According to the SEM images, the COF forms hollow spheres owing to the inside-out Ostwald ripening and attaches on the 3D GCF uniformly (Fig. 5j–o). The as-prepared composite shows good flexibility and mechanical robustness. Meanwhile, the initial conductivity of 1.25 mS cm^−1^ for DAB/GCF increases to 4.17 mS cm^−1^ after applying strain of 70%, which ensures its application as an electrode in flexible SCs. *C*_sp_ of 129.2 F g^−1^ at 0.5 A g^−1^ is obtained, while 68.2% of this value can be retained at 10 A g^−1^.

#### 3.3.2 *In situ* polymerizing conductive polymers in COFs

**Fig. 6:**
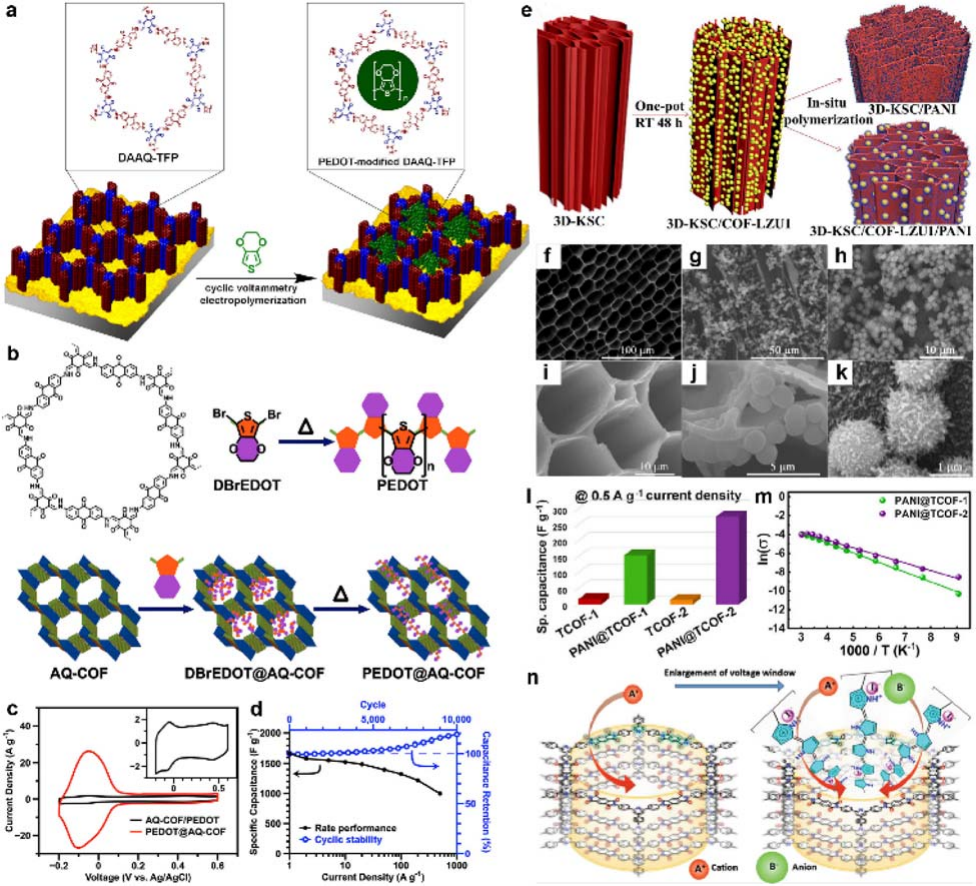
(a) Depiction of the modification of DAAQ-TFP films by electropolymerization of 3,4-ethylenedioxythiophene (EDOT). Reprinted from ref. 54. Copyright 2016, American Chemical Society. (b) Chemical structure of AQ-COF and schematic image for the preparation of PEDOT@AQ-COF. (c) CV curves of AQ-COF/PEDOT and PEDOT@AQ-COF recorded at a scan rate of 5 mV s^−1^. Inset: Enlarged CV curve of AQ-COF/PEDOT. (d) *C*_sp_ of PEDOT@AQ-COF at different current densities, and cycling stability over 10 000 cycles at 50 A g^−1^. Reprinted from ref. 55. Copyright 2019, American Chemical Society. (e) Schematic illustration of synthesizing 3D-KSC/COF-LZU1, 3D-KSC/PANI and 3D-KSC/COF-LZU1/PANI. (f and i) SEM images of 3D-KSC, (g and j) 3D-KSC/COF-LZU1 and (h and k) 3D-KSC/COF-LZU1/PANI. (l) Specific capacitances of TCOFs and PANI@TCOFs at 0.5 A g^−1^ current density. Reprinted from ref. 58. Copyright 2020, Elsevier. (m) Temperature-dependent conductivity profiles of PANI@TCOF-1 and PANI@TCOF-2 (symbols and solid lines denote the experimental and fitted data, respectively). Reprinted from ref. 59. Copyright 2021, Wiley-VCH GmbH. (n) The columnar channel of IISERP-COF30 that interacts with cationic and anionic centers. Reprinted from ref. 60. Copyright 2022, Wiley-VCH GmbH.

Generally, the combination of carbon materials and COFs can only form two separated phases. The conductivity of the composites is still restricted by the thickness of the COF phase. In contrast, the *in situ* polymerization of conductive polymers can at least partially solve this problem owing to the penetrated polymer chains in the COF pores. In 2016, Mulzer *et al.* electropolymerized EDOT (3,4-ethylenedioxythiophene) within the pores of DAAQ-TFP COF (Fig. 6a).^54^ The electrical conductivity is greatly enhanced, which could be evidenced by the sharply reduced solution resistance from 430 Ω to 20 Ω. Moreover, the accessible charge increases from ∼3% for the unmodified DAAQ-TFP film to nearly 100% for its modified counterpart, while the volumetric *C*_sp_ increases more than 17-fold (20 to 350 F cm^−3^). Similarly, Wu *et al.* employed the same strategy but using *in situ* solid-state polymerization (SSP) with DBrEDOT (2,5-dibromo-3,4-ethylenedioxythiophene) as the precursor (Fig. 6b).^55^ The conductivity of PEDOT@AQ-COF is determined to be 1.1 and 1.0 S cm^−1^, according to two- and four-probe methods, respectively. As a result, the peak current density of PEDOT@AQ-COF in the CV test was 15 times that of mechanically mixed AQ-COF/PEDOT, illustrating outstanding electron and mass transfer capability (Fig. 6c). As demonstrated in Fig. 6d, the GCD analysis in a three-electrode system manifests ultrahigh *C*_sp_ of 1663 F g^−1^ at the current density of 1 A g^−1^. Impressively, 60% of this capacitance can be retained at an ultrahigh current density of 500 A g^−1^, while a slight increase of the capacitance is observed after 10 000 cycles at 50 A g^−1^. Such extraordinary rate capability and stability are quite attractive for practical use.

Polyaniline (PANI) is also known as a kind of conductive polymer.^56^ In 2019, Liu *et al.* first synthesized TpPa-COF in the presence of PANI to construct a composite.^57^ Although the cycling stability is good such that 83% of the initial capacitance could be retained after 30 000 cycles, the highest *C*_sp_ is only 95 F g^−1^ obtained at a current density of 0.2 A g^−1^. The accumulation of COF on the network structure could be a possible reason for the low capacitance, which restricts the effect of PANI. Therefore, Peng *et al.* introduced the 3D kenaf stem-derived carbon (3D-KSC) as the template to synthesize COF, and then grew PANI on the surface of the composite *via* chemical oxidation polymerization (Fig. 6e).^58^ The obtained composite (3D-KSC/COF-LZU1/PANI) shows a much higher areal *C*_sp_ of 583.0 mF cm^−2^ at 0.1 mA cm^−2^, compared with 3D-KSC/COF-LZU1 (115.7 mF cm^−2^) and 3D-KSC/PANI (107.5 mF cm^−2^). This huge improvement reveals the synergistic effect of these three components (Fig. 6f–k). Furthermore, the EIS test demonstrates that the *R*_s_ of this electrode is negligible while the charge transfer resistance (*R*_ct_) is only several ohms, illustrating excellent conductivity. In contrast, Patra and Dutta directly introduced PANI into the COF pores *via in situ* chemical oxidation polymerization of aniline.^59^ The composites show significantly improved conductivities of 1.4 × 10^−2^ and 1.9 × 10^−2^ S cm^−1^ for PANI@TCOF-1 and PANI@TCOF-2, while the conductivities of TCOF-1 and TCOF-2 are too low to be detected. Therefore, the highest specific capacitances of PANI@TCOF-1 and PANI@TCOF-2 are 154 and 275 F g^−1^ at 0.5 A g^−1^, much higher than the 10–20 F g^−1^ for TCOF-1 and TCOF-2 (Fig. 6l and m).

In 2022, Haldar *et al.* combined polypyrrole (Ppy) with polyimide (PI) COF (Fig. 6n).^60^ Apart from conductivity improvement, Ppy can also provide redox sites for the PC. Besides, the iodine catalyst for the *in situ* polymerization is able to generate triiodide ions for the redox reaction in the acidic electrolyte. After forming the composite, the overall energy gap obviously reduces from 2.25 eV to 1.58 eV, which is beneficial to enhancing the conductivity to the order of 10^−3^ S cm^−1^. Benefitting from the good conductivity and abundant redox sites of imide, pyridine, pyrrole, and triiodide, the solid-state SC delivers high areal *C*_sp_ of 358 mF cm^−2^ at 1 mA cm^−2^, with excellent cyclic retention of 82% after 6000 cycles.

#### 3.3.3 MOFs@COFs

MOFs@COFs are an emerging type of multifunctional porous material.^61^ By introducing MOFs into the synthetic procedure of COFs, the composite can combine the advantages of each part thus realizing better performances in various application fields than its individual components.

**Fig. 7:**
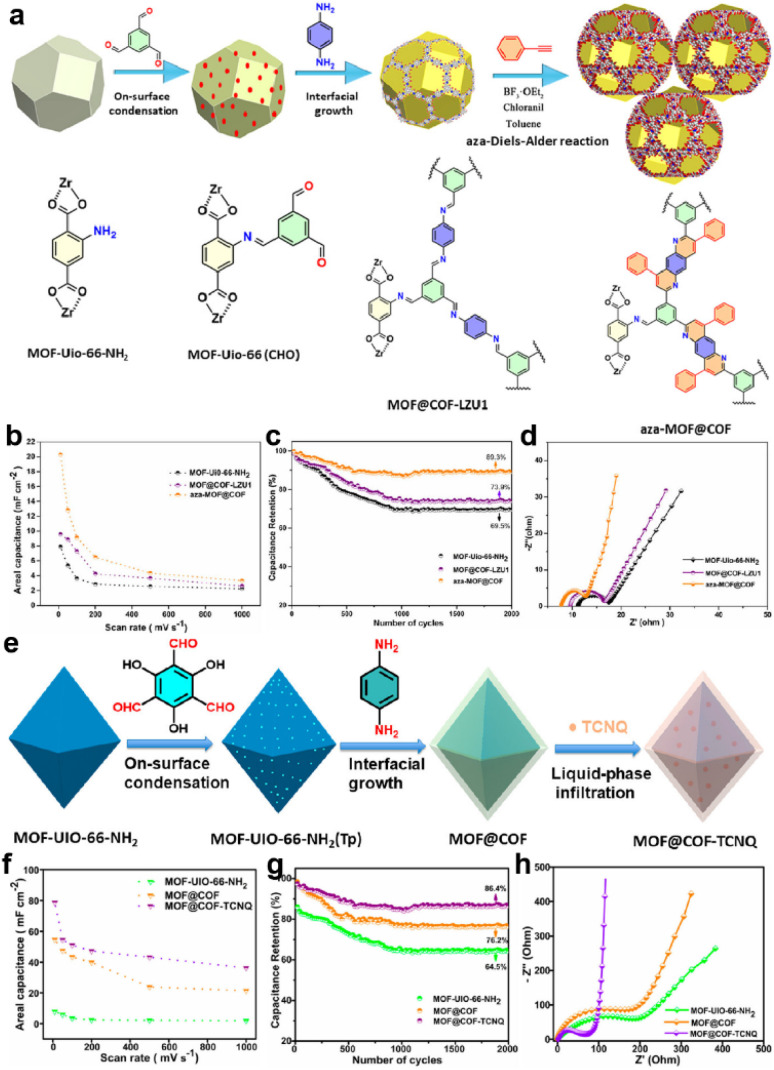
(a) Illustration of the synthetic route of the aza-MOF@COF hybrid structure. (b) Areal capacitance, (c) capacitance retention, and (d) Nyquist electrochemical impedance spectra of MOF-UiO-66-NH_2_, MOF@COF-LZU1, and aza-MOF@COF. Reprinted from ref. 62. Copyright 2020, Wiley-VCH GmbH. (e) Schematic of the synthesis route for MOF@COF-TCNQ hybrids. (f) Areal capacitance, (g) capacitance retention, and (h) Nyquist electrochemical impedance spectra of MOF-UiO-66-NH_2_, MOF@COF, and MOF@COF-TCNQ. Reprinted from ref. 63. Copyright 2021, American Chemical Society.

In 2020, Peng and colleagues introduced UiO-66-NH_2_ into the synthesis of COF-LZU1 as a template (Fig. 7a).^62^ They further underwent an aza-Diels–Alder reaction between phenyl imine and phenylacetylene to form a fused six-membered ring. According to the TEM measurement, the average size of the modified composite (aza-MOF@COF) is ∼230 nm, and the thickness of the COF layer is 30 nm, which favor access to the inner COF layer. The symmetric solid-state SC based on aza-MOF@COF manifests a more than doubled areal specific capacitance compared to the original COF and unmodified MOF@COF (Fig. 7b). Besides, the rate capability, cycling stability, and conductivity of aza-MOF@COF are all superior to them (Fig. 7c and d). Consequently, this post-modification can not only strengthen the backbone for the enhanced stability, but also improve the overall capacitive performance. Soon after, they used another COF (COF-TpPa) as the shell for the MOF@COF composite, and then introduced TCNQ (7,7,8,8-tetracyanoquinodimethane) molecules into the pores of the composite *via* a liquid-phase infiltration method (Fig. 7e).^63^ The incorporated TCNQ mainly serves as the redox-active component and electron transfer promotor. Based on the combination of experimental and theoretical results, the authors claimed that the formation of the composite significantly reduced the *R*_s_, indicating improved electron transfer. Besides, although the MOF@COF composite delivers a reduced quantum areal capacitance compared with the MOF or COF, the introduction of TCNQ can sharply increase this capacitance to ∼3-fold the original value, illustrating the effectiveness of the design concept (Fig. 7f–h).

### 3.4 Structure and morphology control

Apart from the inherent properties of COF-based electrode materials, their structure and morphology are also of great significance for the improvement of SC performance.

#### 3.4.1 Linkage selection

**Scheme 3:**
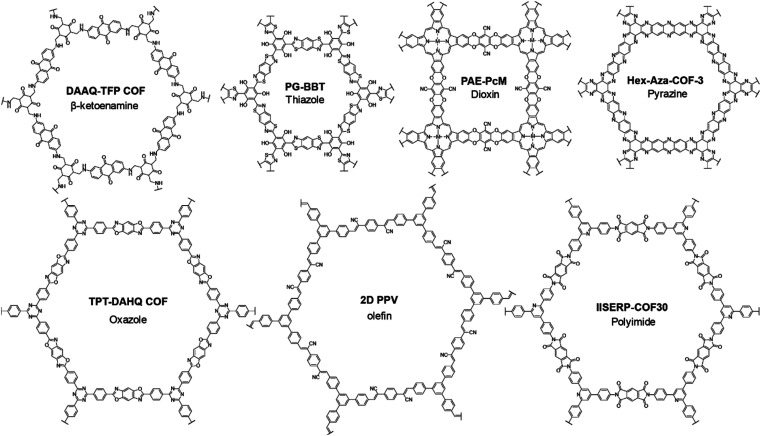
Representative structures of robust COFs for SCs.

As one type of crystalline material, COFs with high crystallinity are achieved by the “error-correction” process during the polycondensation reactions.^64^ However, the reversible condensation reactions also result in the unsatisfactory stability of COFs in the early stage, especially for the borate ester COFs. Although most Schiff-base COFs show good stability under ambient conditions, their structure will collapse under strong acidic or basic conditions, such as aqueous electrolytes for supercapacitors.^65^ Therefore, the stability of the COF should be the first consideration when employed as the electrode material for SCs (Scheme 3).

The β-ketoenamine linkage was the first-employed COF structure for SCs.^29^ The irreversible tautomerism after Schiff base reaction further strengthens the skeleton and improves the tolerance under harsh conditions.^66^ Soon after, Zhuang *et al.* employed an sp^2^-COF constructed through Knoevenagel reaction as the electrode material of SCs.^67^ The fully conjugated carbon backbone manifests excellent thermal and chemical stability even in very strong acid and base. Xu *et al.* synthesized another olefin-linked COF *via* the different Knoevenagel reactions between TFPT (1,3,5-tris-(4-formylphenyl)triazine) and DCTMP (3,5-dicyano-2,4,6-trimethylpyridine).^68^ Besides the high BET SSA of 1003 m^2^ g^−1^, the as-prepared COF manifested outstanding stability, keeping stable in 12 M HCl and NaOH solutions with *T*_d90_ higher than 500 °C. After being mixed with a small portion of SWCNT, the free-standing COF film can be obtained, which was used as an electrode for a micro-SC.

An oxazole-linked COF was firstly synthesized by Pyles *et al.* with moderate crystallinity.^69^ Later in 2018, Waller and colleagues synthesized oxazole- and thiazole-linked COFs *via* a linkage exchange method with excellent crystallinity.^70^ Furthermore, outstanding thermal and chemical stabilities were proven by the thermogravimetry analysis (TGA) and several soaking treatments under harsh conditions. Therefore, oxazole- and thiazole-linked COFs should be promising candidates for SCs. In 2019, Li and co-workers synthesized two thiazole-linked COFs by a one-step method for SCs.^71^ The introduction of hydrogen bonding can further strengthen the skeleton according to the increased decomposition temperature. Besides the stability, the existence of benzobisthiazole can also improve the conductivity of the COF, thus realizing *R*_ct_ lower than 1 Ω. Also this year, El Mahdy *et al.* employed an oxazole-linked COF as the electrode material for SCs.^72^ Combined with a triazine unit, this COF shows integration of PC and EDLC. Only 1.2% capacitive loss is observed after 1850 cycles, illustrating the excellent stability of TPT-DAHQ COF.

Polyimide COFs (PI-COFs) have been demonstrated as promising electrode materials for rechargeable batteries owing to their outstanding stability and abundant redox sites.^73–75^ Similarly, PI-COFs are also a class of promising candidates for SCs. For example, Haldar *et al.* incorporated polypyrrole into the pores of an imide-linked COF, IISERP-COF30.^60^ The COF skeleton remains stable until the temperature reaches 400 °C in a TGA experiment. The several couples of peaks in the CV curve demonstrate the abundant redox sites for PC.

Polyarylether possesses high mechanical robustness and excellent chemical stability. Thus, it is reasonable that dioxin-linked COFs, separately developed by Fang^76^ and Yaghi,^77^ also exhibit excellent stability enabling their utilization in SCs. In 2021, Yang and co-workers integrated dioxin linkage with metallophthalocyanine to construct conductive COFs.^78^ The dioxin linkage can provide a robust skeleton and packing, while metallophthalocyanine can offer the charge transporting channel. The carrier mobilities of COF films are ∼19.4, 3.2, and 0.3 cm^2^ V^−1^ s^−1^ for PAE-PcCo, PAE-PcNi and PAE-PcCu, respectively, determined by the Hall effect measurements. As a result, the quasi-solid-state symmetric SC based on PAE-PcCo achieves areal *C*_sp_ of 19 μF cm^−2^.

Pyrazine-linked COFs have also been used as potential electrode materials in metal-ion batteries because of their high density of redox-active sites and robust structure.^79–81^ These COFs were first applied in SCs by Kandambeth and colleagues.^82^ They synthesized three Hex-Aza-COFs as the negative electrode for asymmetric devices. Among them, Hex-Aza-COF-1 and Hex-Aza-COF-3 deliver excellent thermal stability until 400 °C, while Hex-Aza-COF-2 starts decomposing after 250 °C mainly due to the existence of benzoquinone. Despite their poor crystallinity and low SSA, high specific capacitances of 585 and 663 F g^−1^ at 1 A g^−1^ are acquired by Hex-Aza-COF-2 and Hex-Aza-COF-3, respectively. However, the rate performances of these electrodes are not satisfactory. Recently, Iqbal and co-workers employed a pyrazine-linked HADQ COF as the electrode material for SCs.^83^ The pristine COF was further treated at high temperature for a second polymerization to improve the crystallinity and SSA. The obtained 200-HADQ COF and 250-HADQ COF (respectively treated at 200 and 250 °C) as well as the pristine HADQ COF deliver very high decomposition temperatures all above 600 °C within 2% weight loss. The BET SSA dramatically increases from 411 m^2^ g^−1^ for HADQ COF to 2443 m^2^ g^−1^ for 250-HADQ COF, while the FWHM (full width at half maximum) of the PXRD (powder X-ray diffraction) peak obviously decreases. In a two-electrode coin-cell system using ionic liquid electrolyte, the 250-HADQ COF electrode manifests the highest *C*_sp_ among the three electrodes of 516 F g^−1^ at 0.5 A g^−1^. More excitingly, 67% of this capacitance can be retained at a very high current density of 100 A g^−1^, illustrating the promise of pyrazine-COF for SCs.

#### 3.4.2 Hydrogen bonding

Hydrogen bonds (H-bonds) are a kind of strong non-bonding interaction that plays an unsubstitutable role in life science and also materials science.^84^ In COF structures, hydrogen bonds are also proven to effectively strengthen the skeleton and even influence the redox reactions.

**Fig. 8:**
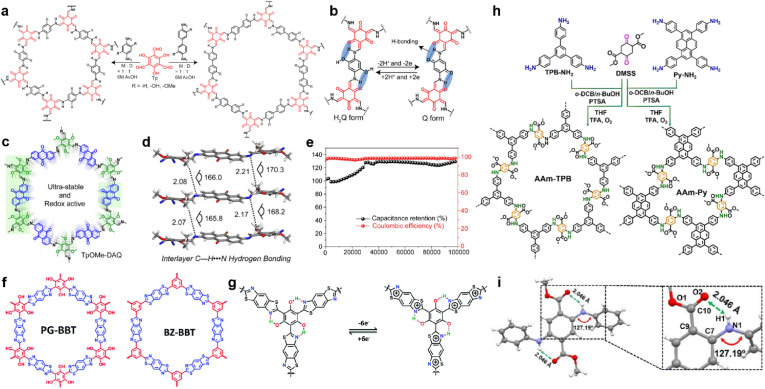
(a) Synthesis of pPa-R_2_ [TpPa-1, TpPa-(OH)_2_, and TpPa-(OMe)_2_] and TpBD-R_2_ [TpBD, TpBD-(OH)_2_, and TpBD-(OMe)_2_] where R = H, OH, and OMe. (b) Proposed H-bonding stabilizing both the hydroquinone (H_2_Q) and benzoquinone (Q). Reprinted from ref. 85. Copyright 2017, American Chemical Society. (c) Structure of TpOMe-DAQ. (d) Interlayer C–H⋯N H-bonding in TpOMe-DAQ. (e) Cycling stability performance (10 mA cm^−2^) of the TpOMe-DAQ electrode. Reprinted from ref. 89. Copyright 2018, American Chemical Society. (f) Structure of PG-BBT and BZ-BBT. (g) Intramolecular hydrogen bonds and charge–discharge process of the benzobisthiazole unit. Reprinted from ref. 71. Copyright 2020, Royal Society of Chemistry. (h) Synthetic routes for arylamine-linked COFs. (i) Intralayer N–H⋯O hydrogen bonding in a model compound of arylamine-linked COF. Reprinted from ref. 93. Copyright 2021, Wiley-VCH Verlag GmbH.

In 2017, Chandra *et al.* prepared several β-ketoenamine-linked COFs using phenylenediamine and benzidine with or without *ortho*-hydroxyl substituents (Fig. 8a).^85^ Owing to the redox interconversion mechanism of hydroquinone (H_2_Q)/benzoquinone (Q) in a buffer solution, a rapid drop of capacitance within a few cycles is expected, caused by the decomposition of benzoquinone. However, the existence of H-bonds between hydroxyl and amine enables the highest specific capacity of 416 F g^−1^ at 0.5 A g^−1^ and long-term cycling stability with up to 88% capacity retention after 10 000 cycles (Fig. 8b).

The tautomerism of β-ketoenamine-linked COFs endows them with excellent stability, but partially sacrifices the crystallinity and conjugation in exchange. Imine-COFs possess excellent crystallinity and conjugation because of the reversibility of Schiff base reaction.^86^ By introducing interlayer H-bonds, their stability can also be significantly enhanced.^87,88^ Following this concept, Halder *et al.* synthesized TpOMe-DAQ through the condensation reaction between DAQ (2,6-diaminoanthraquinone) and TpOMe (2,4,6-trimethoxy-1,3,5-benzenetricarbaldehyde) (Fig. 8c).^89^ The introduction of methoxyl groups can form the interlayer hydrogen bonding with imine groups, thus improving the crystallinity and stability (Fig. 8d). Besides, the free-standing thin sheet of TpOMe-DAQ (200–220 μm) is able to be constructed by a mechanochemical grinding approach, and could be directly utilized as electrodes for SCs. Large areal and gravimetric *C*_sp_ of 1600 mF cm^−2^ and 169 F g^−1^ were achieved from a GCD test in a three-electrode system, respectively. Furthermore, no capacitance loss was investigated even after 100 000 charge/discharge cycles, demonstrating outstanding stability (Fig. 8e). The symmetric solid-state SC could achieve areal *C*_sp_ of 84 mF cm^−2^, accompanied with an energy density of 2.9 μW h cm^−2^ at a power density of 61.8 μW cm^−2^. Haldar *et al.* prepared a family of imine-based COFs using tripyridine–triazine–triamine and trialdehydes with different numbers of hydroxyl substitutes.^90^ Along with the increasing number of hydroxyl groups, the ratio of keto-form tautomerism increases. Meanwhile the PC ratio increases with the hydroxyl number due to the higher affinity of carbonyl groups toward protic electrolyte over the enolic form, as well as more redox sites. Interestingly, the further incorporation of –OH groups also affects the stacking mode of the COF backbone, as IISERP-COF10 (one hydroxyl group) shows AA eclipsed stacking while IISERP-COF11 and IISERP-COF12 (two and three hydroxyl groups) exhibit staggered stacking.

In 2019, Li *et al.* synthesized two thiazole-linked COFs, in which one of them incorporated hydroxyl groups to form H-bonds (Fig. 8f).^71^ Thiazole is well known as an electron transfer unit and widely used in building optoelectronic materials.^91,92^ Introducing benzothiazole in COF can not only enhance the structural stability but also improve the conductivity (Fig. 8g). For instance, the PG-BBT with hydroxyl groups has much higher decomposition temperature than BZ-BBT, although they both exhibit excellent stability under harsh chemical conditions. Interestingly, the hydrogen bonding between the hydroxyl groups and nitrogen atoms of benzothiazole units induces intramolecular proton transfer, thus enabling the extra redox reaction. The EIS test demonstrates very low *R*_ct_ of 1.1 Ω, corresponding to the synergistic effect of H-bond-induced proton-coupled electron transfer and benzothiazole. This resistance goes even lower to 0.6 Ω owing to the improved wettability and activation of the electrode. A highest *C*_sp_ of 724 F g^−1^ is realized in the PG-BBT-based electrode, which can retain 96% of this capacitance after 10 000 cycles.

Yang and colleagues prepared two arylamine-linked COFs through the condensation reaction between arylamines and DMSS (dimethyl succinyl succinate) (Fig. 8h).^93^ Compared with the imine analogues, these two COFs deliver better stability and electrical activity. The dominating origin of this enhancement is the hydrogen bonding between the carbonyl group of the succinate and amine linkage (Fig. 8i). The abundant H-bonds distributed thoroughly on the skeleton can strengthen the structure for excellent stability as well as promoted electron transfer. Eventually, specific capacitive retentions of 74% and 91% are obtained for rate capability at current densities from 1 A g^−1^ to 10 A g^−1^ and cycling stability for 10 000 cycles at 5 A g^−1^, respectively.

The NKCOFs reported by Yang *et al.* also involve the H-bonds within the skeleton.^44^ During the discharging process, the protons in the acidic electrolyte convert azo bonds into single bonds thus enabling the proton-coupled electron transfer (PCET) along with the 1D channel within the COF. The authors proved that this proton conducting progress belongs to the Grotthuss mechanism with proton hopping within the hydrogen-bonded network.

#### 3.4.3 Morphology control

**Fig. 9:**
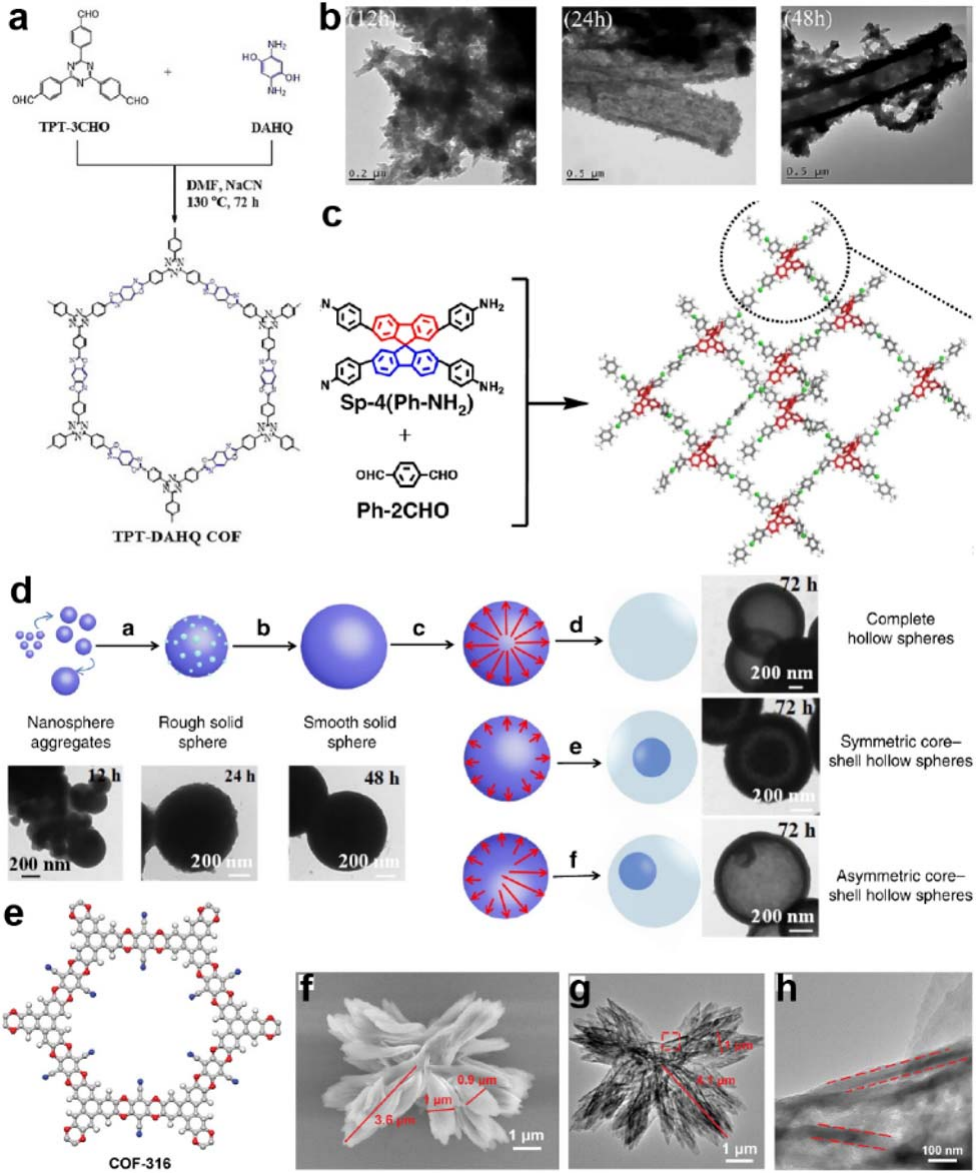
(a) Synthesis of TPT-DAHQ COF. (b) TEM images of the hollow microtubular TPT-DAHQ COF having sponge-like shells, measured after reaction times of 12, 24, and 48 h. Reprinted from ref. 72. Copyright 2019, Wiley-VCH Verlag GmbH. (c) Synthesis of 3D-Sp-COFs. (d) Self-templated hollowing evolvement process of 3D-Sp-COFs *via* the Ostwald ripening mechanism. Reprinted from ref. 94. Copyright 2020, Springer Nature Limited (Creative Commons CC BY). (e) Chemical structure, (f) SEM image, (g) TEM image, and (h) enlarged TEM image (the red frame in (g)) of COF-316. Reprinted from ref. 32. Copyright 2021, Wiley-VCH GmbH.

Large surface area is one of the inherent advantages of COFs that enables the fast ion and mass transportation in the electrode.^35^ However, direct utilization of bulk COFs with random orientation may obstruct the 1D channel originating from the stacked pores. Therefore, morphology control is of great importance for the improvement of overall device performance. For instance, El Mahdy *et al.* synthesized an oxazole-linked COF in 2019 (Fig. 9a).^72^ According to the SEM and TEM tests in Fig. 9b, the COF can form ribbon-like morphology through self-assembly, and then undergo an Ostwald ripening process to form hollow microtubes. As a result, TPT-DAHQ COF achieves a very high BET SSA of 1855 m^2^ g^−1^. As the authors claim, the numerous pore sizes and large surface area provided by the tubular structure enable good electrode–electrolyte contact and fast ion diffusion.

Soon after, Liu *et al.* constructed an imine-linked 3D-COF (Fig. 9c).^94^ It is found that when extending the reaction time to over 72 h, the COF gradually forms a hollow spherical morphology controlled by the Ostwald ripening process (Fig. 9d). Meanwhile, the SSA of the COF remarkably increases along with the extension of the reaction time. This enhancement is apparently reflected in the SC performance. Specific capacitances of 45, 64, 86, 100, 180, and 251 F g^−1^ at a current density of 0.5 A g^−1^ were achieved for 3D-Sp-COF@6 h, 3D-Sp-COF@12 h, 3D-Sp-COF@24 h, 3D-Sp-COF@48 h, 3D-Sp-COF@72 h, and 3D-Sp-COF@96 h, respectively, illustrating the significant impact of morphology on the SC performance. Wang *et al.* also reported an Ostwald ripening driven assembly of a dioxin-linked COF to form a more complicated hollow flower morphology (Fig. 9e–h).^32^ The hollow structure can directly achieve a 19% increase of the capacitance compared with the bulk COF, illustrating the enhancement of the accessibility of the hollow structure.

**Fig. 10:**
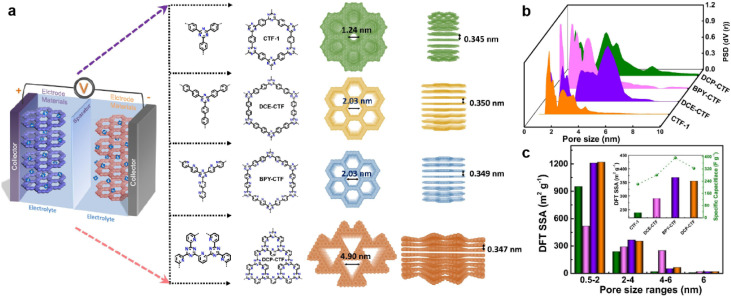
(a) Schematic diagram of supercapacitor configuration together with design and synthesis of CTF-1, DCE-CTF, BPY-CTF and DCP-CTF, including the top and side views from left to right. (b) The pore size distribution calculated by QS-DFT for CTF-1, DCE-CTF, BPYCTF, and DCP-CTF. (c) DFT SSA distribution of different pore size ranges, the inset is DFT SSA of CTFs derived from pores with size range of 2–4 nm *versus* specific capacitance. Reprinted from ref. 95. Copyright 2022, Elsevier.

The pore size also plays a key role in enhancing the capacitive performance. Zhang *et al.* prepared a family of CTFs with different pore sizes distributed from 1.2 to 5.0 nm (Fig. 10a).^95^ The BPY-CTF with pore size distribution of 1.5–3.0 nm shows the highest *C*_sp_, while DCP-CTF with similar effective SSA but larger pore size distribution (1–4 nm) exhibits slightly lower *C*_sp_. However, the CTF-1 with micropores and DCE-CTF with much larger pores (mainly distributed at 4.9 nm) show much lower capacitance (Fig. 10b and c). As the pore size should match the size of electrolyte molecules, some other works claim that the micropore with a diameter of 1.0–2.0 nm is more appropriate.^45,96^

**Fig. 11:**
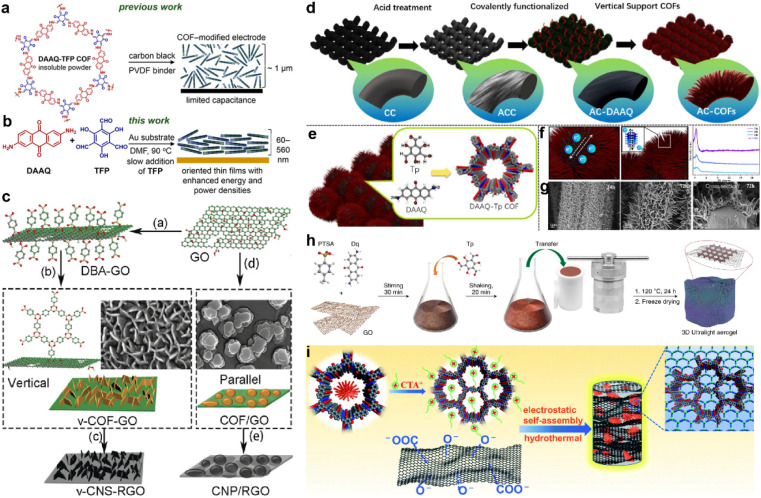
(a) Structure of the hexagonal subunit of the DAAQ-TFP COF, which was previously prepared as a slurry, mixed with carbon black and a PVDF binder, for supercapacitor applications. (b) Solvothermal growth of the DAAQ-TFP COF as an oriented thin film on Au electrodes. Reprinted from ref. 97. Copyright 2015, American Chemical Society. (c) Schematic illustration of the preparation of v-COF–GO and COF/GO to produce carbon nanosheets oriented vertical or parallel to the rGO surface. Reprinted from ref. 99. Copyright 2018, Wiley-VCH GmbH. (d) Schematic diagram of the functionalized AC-COFs. (e) Synthesis and structural characterization of COFs. (f) Schematic illustration of electron transport in AC-COFs and PXRD patterns of the AC-COFs at different times. (g) SEM and cross-section images of AC-COFs at different time intervals. Reprinted from ref. 100. Copyright 2022, American Chemical Society. (h) Scheme of the synthetic procedure for the preparation of the COF/rGO aerogel. Reprinted from ref. 101. Copyright 2020, Springer Nature Limited (Creative Commons CC BY). (i) Scheme of the synthesis procedure for the DAAQ-COFs/GA composite. Reprinted from ref. 102. Copyright 2021, Royal Society of Chemistry.

Apart from morphology and pore size, the orientation of the COF structure also influences the accessibility of redox sites and capacitor performance. The first COF-based SC only achieved the *C*_sp_ of 48 F g^−1^ due to the poor accessibility to the redox sites (3%). To optimize the orientation of COF film, the COF film was slowly grown on an Au substrate and realized about 20% access to the redox sites (Fig. 11a and b).^97^ Besides the orientation, the well-controlled thickness of the COF film (∼180 nm) also contributes to the increase of the performance as a thick film will hinder the penetration of the electrolyte.

Several groups combined the COF with other carbon materials *via in situ* growth to improve the conductivity. Generally, the COFs grow on the graphene or carbon nanotubes in the face-to-face direction as the carbon materials also act as the template.^98^ But the too-thick COF layer also can lead to the difficulty in penetration of electrolyte similar to the DAAQ-TFP on an Au substrate. In contrast, Sun and colleagues grew the 2D COF on GO in the perpendicular direction.^99^ The DBA (benzene-1,4-diboronic acid) was firstly grafted on the modified GO surface to fix the perpendicular direction. Then the *in situ* polycondensation took place to grow the COF vertically (Fig. 11c). Considering the poor stability of the borate ester COF, they further pyrolyzed the COF to carbon nanosheets. The as-prepared v-CNS-RGOs show a combination of PC and EDLC, and exhibit excellent rate capability and cycling stability when employed as the electrode of an SC.

Recently, He *et al.* grew DAAQ-Tp COF on carbon cloth (CC).^100^ The morphology of COF was controlled by a three-step procedure. The CC was firstly treated by strong acid and oxidant to form oxygen-rich functional groups (AC). Then, the DAAQ molecules were anchored on these sites *via* covalent bonds to control the growth position of COF. Finally, the Tp and TAAQ precursors could be added for the *in situ* condensation reaction (Fig. 11d). Interestingly, the COF eventually becomes vertical tentacle-like arrays on the surface of the carbon cloth fiber, and further forms interlaced conductive networks (Fig. 11e–g). Even when the composite is applied in the symmetric flexible solid-state SC, the areal *C*_sp_ of 715 and 552 mF cm^−2^ can be obtained at current densities of 0.5 and 7 mA cm^−2^, respectively. Such high capacitance and excellent rate capability demonstrate the outstanding charge migration and ion diffusion enabled by the well-controlled morphology.

Morphology control is also significant for the substrate of COF-based composites. Li *et al.* developed a COF/rGO aerogel based on the *in situ* preparation of the well-known TpDq-COF in the presence of GO (Fig. 11h).^101^ The as-prepared composite exhibits a hierarchical porous 3D sponge-like structure with ultralight weight, good mechanical strength and high conductivity. Combined with the good adsorption performance of COF/rGO aerogel, excellent accessibility of redox sites within the composite to the electrolyte is predictable. As expected, the direct use of this composite as the electrode achieves very low *R*_s_ and *R*_ct_ according to the EIS test. Meanwhile, *C*_sp_ of 269 F g^−1^ at the current density of 0.5 A g^−1^ is realized, and 83% of *C*_sp_ can be retained at a 20-fold current density of 10 A g^−1^, illustrating outstanding rate performance among COF-based electrode materials. Soon after, An *et al.* developed a similar COF/GO aerogel.^102^ Differently, they firstly synthesized the COF with a flower-like morphology and then combined it with GO to form COF/GO aerogel *via* an electrostatic self-assembly procedure (Fig. 11i). Due to the hierarchical structure of COF, the BET SSA of the composite is much higher than in the previous work (425.3 m^2^ g^−1^*vs.* 246 m^2^ g^−1^). In the three-electrode system with acidic electrolyte, high *C*_sp_ of 378 F g^−1^ is achieved at the current density of 1 A g^−1^. Impressively, 81.5% of the initial capacitance can be retained at a high current density of 70 A g^−1^, and a cyclic retention of 87.8% after 20 000 cycles at a current density of 15 A g^−1^ is obtained. Even in an asymmetric supercapacitor (ASC) device, a highest *C*_sp_ of 378 F g^−1^ at 1 A g^−1^, 75% retention of rate capability at 10 A g^−1^, and cyclic retention of 88.9% after 20 000 cycles at 5 A g^−1^ can be realized, indicating the success of the proposed strategy.

### 3.5 Other strategies

In recent years, researchers have not been content with the former traditional methods for performance enhancement. Along with revealing the working mechanism in depth, some other strategies were proposed and some examples are introduced in this part.

#### 3.5.1 Introducing heteroatoms

**Fig. 12:**
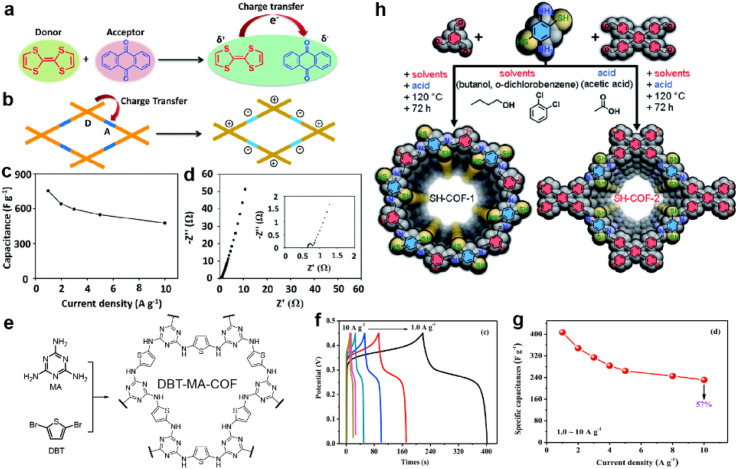
Illustration of charge transfer (a) in tetrathiafulvalene and *p*-benzoquinone compounds and (b) in the donor–acceptor COF. (c) Capacitance *vs.* current density and (d) Nyquist plot (the inset is the magnified Nyquist plot) of the TTF-COF1 electrode in a three-electrode system. Reprinted from ref. 105. Copyright 2020, Royal Society of Chemistry. (e) Synthetic route and structure of DBT-MA-COF. (f) GCD curves and (g) Specific capacitances at various current densities of DBT-MA-COF. Reprinted from ref. 106. Copyright 2021, Elsevier. (h) Synthetic pathway of SH-COF-1 and SH-COF-2. Reprinted from ref. 107. Copyright 2022, Royal Society of Chemistry.

Sulfur-containing groups often possess excellent electric activity which can be introduced to construct organic conductive materials, such as Cu-BHT MOF^103^ and tetrathiafulvalene^104^ (TTF). Thus, the improvement of the conductivity can be expected when incorporating sulfur-containing groups into COFs. In 2020, Li *et al.* constructed a donor–acceptor (D–A) type COF for SC application (Fig. 12a).^105^ Apart from improving the conductivity, the TTF unit can also act as a donor unit to form a D–A structure with an anthraquinone unit to promote the charge transfer (Fig. 12b). As a result, this TTF-COF1 shows high *C*_sp_ of 758 and 752 F g^−1^ from a CV test at 10 mV s^−1^ and GCD test at 1 A g^−1^, respectively (Fig. 12c). Notably, the *R*_ct_ of TTF-COF1 is only several hundreds of mΩ according to the electrochemical impedance spectroscopy (EIS) test in Fig. 12d, indicating the success of the design concept.

Li *et al.* coupled thiophene with triazine *via* an amine linkage to increase the content of the heteroatoms (Fig. 12e).^106^ Due to the irreversibility of the Buchwald–Hartwig reaction between bromide and amine compounds, the obtained COF exhibits poor crystallinity and relatively low SSA. Nevertheless, the existence of p–π conjugation and intralayer C–H⋯N hydrogen bonding between the C–H of thiophene and N of triazine endow the COF with high conductivity of 2.94 × 10^−2^ S cm^−1^, as well as low *R*_ct_ and *R*_s_, with the assistance of 15% acetylene black. Specific capacitances of 407 and 90 F g^−1^ are achieved at 1 A g^−1^ in a three-electrode system and asymmetric capacitor, respectively (Fig. 12f and g).

In 2022, Pakulski *et al.* decorated thiol groups on the COF backbone as the redox active groups (Fig. 12h).^107^ Compared with the counterparts without thiol groups (59 mF cm^−2^ for COF-1 and 31 mF cm^−2^ for COF-2 at 0.5 mA cm^−2^), the thiol decorated COFs deliver a more than doubled areal specific capacitance (118 mF cm^−2^ for SH-COF-1 and 74 mF cm^−2^ for SH-COF-2 at 0.5 mA cm^−2^). Besides the higher capacitance of SH-COF-1, it possesses larger SSA, an optimal pore size, and more thiol units per cavity, further illustrating the effectiveness of thiol groups for capacitive enhancement.

In 2021, Qiu *et al.* employed a phosphorus-containing imine-linked COF (Phos-COF-1) as the electrode material for an SC.^108^ After combining with conductive carbon, the electrode manifests low *R*_s_ and *R*_ct_ of 1.15 and 3.76 Ω, respectively. A highest *C*_sp_ of 100 F g^−1^ is achieved but with poor rate capability. The diffusion controlled redox reactions confirm the contribution of triphenylphosphine.

#### 3.5.2 Introducing metal atoms

MOFs have been applied earlier in supercapacitors.^12,109^ Owing to the metal coordination, some MOFs deliver outstanding crystallinity and conductivity. Based on this consideration, metal atoms could also be inserted into COFs to enhance the conductivity and crystallinity.

**Fig. 13:**
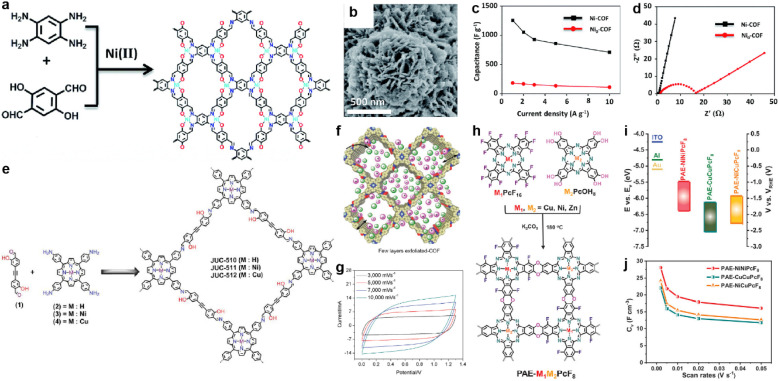
(a) Synthesis and (b) SEM image of Ni-COF. (c) Capacitance and (d) Nyquist plots of Ni-COF and Ni_0_-COF. Reprinted from ref. 110. Copyright 2019, Royal Society of Chemistry. (e) Synthesis of the COF series (JUC-510, JUC-511, and JUC-512). (f) Representative diagram of the charge storage behavior of exfoliated COFs (e-COFs). (g) CV curves of the e-JUC-511 capacitor cell at scan rates of 3000–10 000 mV s^−1^. Reprinted from ref. 111. Copyright 2020, Wiley-VCH Verlag GmbH. (h) Synthesis of PAE-M_1_M_2_PcF_8_ (M_1_, M_2_ = Ni, Cu, Zn) with dioxin linkages. (i) CB and VB of PAE-NiNiPcF_8_, PAE-CuCuPcF_8_, and PAE-NiCuPcF_8_. (j) Specific volumetric capacitances of PAE-M_1_M_2_PcF_8_ calculated from CV curves at different scan rates. Reprinted from ref. 112. Copyright 2022, Wiley-VCH GmbH.

In 2019, Li *et al.* prepared a salphen-containing COF and linked it with nickel ions simultaneously (Fig. 13a).^110^ Comparing with the counterpart without nickel ions (Ni_0_-COF), Ni-COF exhibits extremely enhanced conductivity of 1.3 × 10^−2^ S cm^−1^ in powder form (8.4 × 10^−6^ S cm^−1^ for Ni_0_-COF). When coated on a quartz substrate to form a film, even higher conductivity of 1.2 S cm^−1^ can be realized. Furthermore, the *R*_ct_ of Ni-COF is much lower than that of Ni_0_-COF according to the EIS analysis. As a result, the Ni-COF based electrode manifest outstanding *C*_sp_ of 1478 F g^−1^ at the current density of 0.5 A g^−1^. In contrast, Ni_0_-COF only shows *C*_sp_ of 204 F g^−1^ at the same current density (Fig. 13b–d). Considering the negligible capacitive contribution of nickel foam, the authors concluded that the Ni(ii) has an integrated effect of promoting the hydroquinone to benzoquinone transformation, as well as accelerating electron transport by the highly conjugated planar structure and the high electrical conductivity. Besides, the vertically distributed nanosheet morphology of Ni-COF should enhance the accessibility of the redox sites (Fig. 13b).

In fact, the metal ion isolated from the linkage can also affect the conductivity of the COF. Yusran and co-workers synthesized three COFs between 4,4′-(1,2-ethynediyl)bis-2-hydroxybenzaldehyde and tetrakis(4-aminophenyl)porphyrin with or without metal coordination (Fig. 13e).^111^ After the exfoliation to form nanosheets, these e-COFs show similar morphology (Fig. 13f). However, even the e-JUC-510 without metal ions possesses the largest SSA, it exhibits the lowest *C*_sp_ of 4.17 mF cm^−2^ at scan rate as high as 1000 mV s^−1^ and largest *R*_s_ of 60 Ω. In contrast, corresponding values of 5.46 mF cm^−2^ and 10.8 Ω are obtained for e-JUC-511 (nickel coordinated), and 5.85 mF cm^−2^ and 15.5 Ω for e-JUC-512 (copper coordinated). Such results prove the importance of metal centers (Fig. 13g). Li *et al.* further studied the impact of different types of metal centers (Fig. 13h).^112^ A series of dioxin-linked COFs consisting of electrophilic and nucleophilic phthalocyanines (Pcs) were synthesized with different combinations of metal centers. The crystallinity and morphology of these bulk COFs are similar. But interestingly, the conjugated COFs with different metal centers display a significant difference in electronic properties. For instance, the energy level of the conductive band and valence band of the bulk COFs gradually decreases when the metal center changes from PAE-NiNiPcF_8_ to PAE-NiCuPcF_8_, and then to PAE-CuCuPcF_8_ (Fig. 13i). This is due to the smaller resonance energy with neighboring units of NiPc, thus decreasing the energy barrier of the out-of-plane charge transfer transition. The authors claim that the proximity effect might have a significant impact on the electronic structure of COFs. By mixing with exfoliated graphene to afford the electrode, the micro-supercapacitor based on PAE-NiNiPcF_8_ shows the highest volumetric *C*_sp_ of 28.1 F cm^−3^ at 2 mV s^−1^. Excellent cycling stability with 98.1% retention after 10 000 cycles at 50 mV s^−1^ was also achieved (Fig. 13j).

In 2022, stibium (Sb) was introduced into the COF backbone by Qiu *et al.*^113^ In contrast to the analogue containing nitrogen in the same main group with planar structure (IISERP-COF2 (ref. 114)), this Sb-COF exhibits twisted configuration owing to the lone pairs of Sb atoms.^115^ Compared with a similar COF having a phosphorus center (Phos-COF^116^), Sb-COF shows a cylindrical-like morphology with a much smaller average diameter of ∼100 nm (5–15 μm for Phos-COF). Such small size is beneficial to the large BET SSA (969 m^2^ g^−1^) and the ease of accessing active sites. Indeed, 42% of the triphenylstibine units can be reached during the electrochemical process, much higher than that of Phos-COF (12%). Besides, the percentage of accessibility gradually increases to 90% after 60 000 cycles. The highest *C*_sp_ of 260 F g^−1^ is obtained at 2 A g^−1^, while 72% of this value can be retained at a high current density of 20 A g^−1^, suggesting good rate capability.

#### 3.5.3 Electrolyte management

The electrolyte plays a crucial role in the performance of SCs. It directly affects their capacitive behavior and overall efficiency. The requirements of SCs on electrolytes include wide potential window, high ionic conductivity, good electrochemical stability, low volatility, and low cost.^117^ The optimal electrolyte can increase the charge storage capacity of the COF, improve the charge transfer at the electrode/electrolyte interface, and ensure the long-term stability of the SCs. Although the fabrication of individual components of a capacitor (*e.g.*, electrode material, electrolyte) is relatively simple, compatibility between the pore size and structure of the electrode material and the size of the electrolyte ions is important to improve their combined effect.

The electrolytes employed in the SCs can be basically categorized into three types: aqueous electrolytes, organic electrolytes and hybrid electrolytes. Aqueous electrolytes are usually based on inorganic acids, bases, or salts (*e.g.*, H_2_SO_4_, KOH).^118^ This class of electrolytes are commonly used in SCs due to their low cost, high ionic conductivity, environmental friendliness, high power density, and high capacitance. However, their narrow electrochemical window limits the operating voltage of SCs and induces their limited energy density. In addition, water-based electrolytes are prone to evaporation, which can lead to a reduction in the volume of the electrolyte, thereby reducing the overall performance of the SC. Compared with aqueous electrolytes, organic electrolytes are more demanding in terms of preparation process and conditions. Generally, organic electrolytes (*e.g.*, acetonitrile or propylene carbonate with dissolved salts, such as LiPF_6_) have a wider electrochemical window which allows high energy densities. However, the relatively low ionic conductivity of organic electrolytes limits the power density of SCs. In addition, organic electrolytes are often flammable and not as environmentally friendly as aqueous electrolytes. Taking advantage of both aqueous and organic electrolytes, hybrid electrolytes often show an improved voltage window and ionic conductivity owing to the complementary effect of different components. The ease of preparation and excellent safety enable their utilization in flexible SCs.^119^

**Fig. 14:**
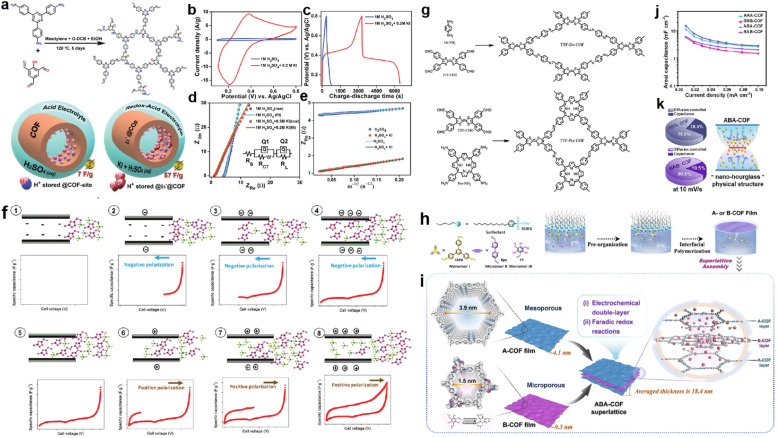
(a) Formation of IISERP-COF25 and illustration of the capacitance enhancement in a COF-derived charge-storage system obtained by the inclusion. (b) CV plots and (c) GCD profile of the composite electrode measured using 1 M H_2_SO_4_ and 1 M H_2_SO_4_ + 0.2 M KI as electrolyte. (d) Nyquist plots from the AC-impedance measurements of the composite and (e) plots of the *Z*_Re_*versus ω*^−1/2^ of the composite measured using the three-electrode assembly. Reprinted from ref. 121. Copyright 2021, Wiley-VCH Verlag GmbH. (f) Mechanism of ionic liquid rearrangement in micropores of TTF-Por COF based EDLC supercapacitors. (g) Schematic of the synthesized TTF-Da COF and TTF-Por COF. Reprinted from ref. 122. Copyright 2023, Wiley-VCH Verlag GmbH. (h) Schematic synthesis of A- and B-COF films using the SMAIS method. (i) Illustration of assembling A- and B-COF films in the ABACOF superlattice. (j) *C*_sp_ of AAA-, BBB-, ABA- and BAB-COF superlattices. (k) Pie chart of ABA- and BAB-COF superlattices at 10 mV s^−1^ and an illustration of the ABA-COF superlattice's internal “nano-hourglass” physical structure. Reprinted from ref. 123. Copyright 2023, Springer Nature (CC BY 4.0).

Adding a redox additive in the electrolyte can profit the capacitive enhancement, which has been evidenced in other systems such as MOF-based SCs.^120^ In 2021, Kushwaha *et al.* introduced KI as a redox additive to improve the device performance of a COF-based capacitor.^121^ The selected COF (IISERP-COF25) has carbonyl units after tautomerism in the presence of protons that can interact with polyiodide species, in which the I_3_^−^ plays the dominant role (Fig. 14a). According to the CV tests, the current response activity of the three-electrode system sharply enhances after the addition of KI in the electrolyte, while the CV curves at different scan rates reveal that the charge storage mechanism becomes more diffusion controlled. Meanwhile, both the *R*_s_ and *R*_ct_ decrease significantly, indicating the enhanced charge transfer induced by KI. As a result, the *C*_sp_ of the three-electrode system increases from 7 to 57 F g^−1^ after the addition of KI (Fig. 14b–e).

Very recently, Chatterjee *et al.* constructed two TTF-based COFs with different charge transfer capabilities (Fig. 14g).^122^ The authors found that only in the two-electrode system using IL electrolyte did the TTF-based electrodes show a couple of small peaks in the CV curves. After carefully investigating the mechanism, the authors claimed that charge storage was dominated by surface charge density and pore size. Thus, by carefully selecting the combination of the COF with negative surface charge density, IL electrolyte, and controlling the charge transfer and polarization, an improvement of the total device performance can be acquired (Fig. 14f).

#### 3.5.4 Mixed COF structure

Recently, Xu and co-workers proposed a “nano-hourglass” structure to promote charge accumulation for increase of capacitance.^123^ They firstly prepared few-layered films of A-COF and B-COF, and then assembled them in certain orders such as ABA and BAB (Fig. 14h and i). Although the capacitive type is dominated by the outer layer, the ABA electrode shows higher *C*_sp_ than the AAA electrode (Fig. 14j). Such enhancement can be ascribed to the “nano-hourglass”, describing the charge accumulation within this nanostructure (Fig. 14k). The outer layer of A-COF with mesopores can trap electrolyte molecules and transfer them to the B-COF layer for the fully performed redox reactions. Meanwhile, the micropores of the B-COF layer are sandwiched by the mesopores of A-COF, which protect them from collapse to realize enhanced rate capability.

#### 3.5.5 COF-derived carbons

COFs usually possess high specific surface area, conjugated units, and controllable heteroatom doping, but with limited conductivity. Therefore, it is feasible to pyrolyze COFs to form corresponding carbon materials. The conjugated structure would ensure the high ratio of graphene-like carbon, thus providing high conductivity, while the high SSA and heteroatoms can be inherited for the efficient redox capacitance. In 2016, Feng *et al.* pyrolyzed the fully conjugated sp^2^-COF into porous carbon without the utilization of a template.^67^ The 2DPPV-800 exhibits the highest capacity of 334 F g^−1^ at 0.5 A g^−1^ compared with other carbon materials pyrolyzed at different temperatures. Notably, no capacitance loss was observed after 10 000 charging/discharging cycles, indicating outstanding cycling stability.

Later, Jiang and co-workers coated an imine-COF on PPZS (polycyclotriphosphazene-*co*-4,4'sulfonyldiphenol) spheres with different weight ratios to form a series of core–shell structure materials.^124^ After pyrolysis at 900 °C, the TAPT–DHTA–COF_0.1_@PPZS_900_ (0.1 ratio of COF) shows a complete and thinnest shell structure, accompanied with the best capacitive performance. The core–shell structure manifests a synergistic effect of facilitating electron conduction and ion transport according to CV and EIS curves.

## 4. SC device applications

This part will discuss the application of COFs in various types of SCs, such as micro-SCs, symmetric SCs, asymmetric SCs, and flexible SCs. The first section explores different COFs for symmetric SCs, highlighting their cycling stability, power density, energy density, and specific capacitance. The second section focuses on asymmetric SCs using COFs combined with different materials for improved performance. The third section addresses flexible SCs, showcasing COFs' benefits in providing mechanical flexibility and stability for wearable electronic devices. Lastly, the fourth section discusses micro-SCs, emphasizing their potential for small-sized, high-energy, and long-life energy storage.

### 4.1 Symmetric SCs

Symmetrical supercapacitors (SSCs) adopt symmetrical device design, where the negative and positive electrodes are matched with identical materials of identical quality, which have fast charging/discharging ability, high power density, and no memory effect. The simple structure and ease in promotion of SSCs facilitate their great application potential and development space in the field of green energy.

In 2018, Halder *et al.* constructed a solid-state SSC using a redox active and hydrogen bonded COF to achieve a high areal capacitance of 84 mF cm^−2^.^89^ The device exhibits energy and power densities of 2.9 μW h cm^−2^ and 61.8 μW cm^−2^ using 2 M aq. H_2_SO_4_/PVA gel as an electrolyte, respectively. This hydrogen-bonded COF has excellent stability and redox activity, so it can be directly used as an electrode material for SCs. In addition, doped COFs are common electrode materials. Among the dopant-modified COF materials, there are many studies based on CTF. Wu *et al.* employed 2,6-pyridinedicarbonitrile to construct porous covalent triazine-based frameworks containing repeating pyridine units (p-CTFs) *via* a trimerization reaction followed by annealing at a certain temperature.^125^ The authors found that the annealing of the synthesized p-CTF at higher temperatures yielded a larger SSA of the product, which should be a significant factor determining the energy storage properties of the material. The authors assembled two-electrode SSCs (button cell) using p-CTF-800 annealed at 800 °C as the electrode material and 1 M H_2_SO_4_ as electrolyte. These devices could achieve a *C*_sp_ of 245.7 F g^−1^ at a current density of 0.2 A g^−1^. Meanwhile, the devices demonstrate the highest energy density of 6.91 W h kg^−1^ at a power density of 50 W kg^−1^ and the highest power density of 2700 W kg^−1^ at an energy density of 5.35 W h kg^−1^. In addition, the devices achieve 95.5% capacitance retention after 10 000 charge/discharge cycles.

In another report, Gao *et al.* synthesized a family of CTFs with fluorine/chlorine functionalization, where the final fluorinated CTF (FCTF) exhibits superior electrochemical properties.^126^ The high degree of fluorination of the CTFs resulted in superior electrochemical properties. The fluorine atoms broaden the interlayer space of the CTFs, which results in a larger SSA and more active sites for the FCTFs. Meanwhile, the fluorination of FCTF is helpful in improving the stability of the material structure. The SSC assembled with FCTF as electrodes and 1 M H_2_SO_4_ as electrolyte demonstrates a specific capacitance of 173 F g^−1^ at 1 A g^−1^. The device shows a high energy density of 54 W h kg^−1^ and a maximum power density of 14 060 W kg^−1^. In addition, the device delivers excellent cycling stability with 98% of the initial capacitance retained after 10 000 charge/discharge cycles at 5 A g^−1^. On the basis of the above work, Gao *et al.* prepared a series of F/N-rich porous carbon (FNC) with large SSA and a large number of active sites by adjusting the carbonization temperature using fluorine-functionalized CTF (FCTF) as a precursor.^127^ Among all the prepared FNCs, the FNC obtained at 700 °C (FNC-700) shows excellent electrochemical performance. The assembled SSC using FNC-700 as electrodes and 1 M KOH as electrolyte achieves a maximum *C*_sp_ of 115 F g^−1^ at 1 A g^−1^. Besides, the maximum energy density is 31.4 W h kg^−1^ while the maximum power density is 7000 W kg^−1^.

**Fig. 15:**
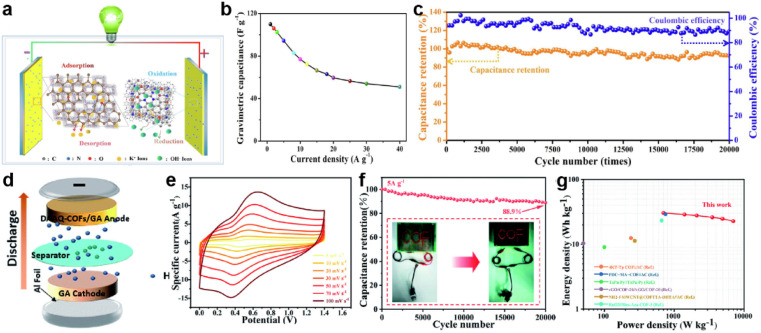
(a) Schematic structure, (b) specific capacitance, and (c) cycling stability and coulombic efficiency of COF-900-based SSCs. Reprinted from ref. 125. Copyright 2023, Elsevier. (d) Schematic of the DAAQ-COFs/GA//GA ASC device. (e) CV curves of the ASC at different scan rates. (f) Stability test at 5 A g^−1^ for 20 000 cycles (the inset shows a digital photograph of the LED powered by the ASC). (g) Ragone plot of the ASC. Reprinted from ref. 102. Copyright 2021, Royal Society of Chemistry.

Additionally, Ma *et al.* developed several nitrogen/oxygen atom-doped mesoporous/microporous carbon frameworks from imine-based and triazine-based COFs *via* high-temperature carbonization.^128^ Among them, the COF-900 based SSC provided an ideal energy density of 15.28 W h kg^−1^ at 352.5 W kg^−1^, and maintained 7.07 W h kg^−1^ even at 7.72 kW kg^−1^ (Fig. 15a). Fig. 15b demonstrates the achievement of specific capacitances of 110 and 51 F g^−1^ at current densities of 1 and 40 A g^−1^, respectively. More importantly, after 20 000 charge/discharge cycles, the device's capacitance retention still remains at 92.6% of its initial value (Fig. 15c). This demonstrates the excellent cycling stability and reversibility of the device. More specific is the thiol-modified COF. Pakulski *et al.* synthesized thiol-decorated COFs (SH-COFs) by condensation reaction between TBA (benzene-1,3,5-tricarboxaldehyde) or TFPB (1,2,4,5-tetrakis-(4-formylphenyl)benzene) and DABDT (2,5-diaminobenzene-1,4-dithiol).^107^ The SH-COF-1 and SH-COF-2 based SSCs using Et_4_NBF_4_/MeCN as electrolyte have area capacitance of 40 mF cm^−2^ and 16 mF cm^−2^, respectively.

Other researchers have improved the electron transfer and ion diffusion capabilities of materials by compositing COF with carbon materials. Wang *et al.* prepared COF/rGO-*x* with different COF mass loadings by mixing reduced graphene oxide (rGO) and TaPa-Py COF.^129^ Subsequently, the thin-film electrodes are prepared with COF/rGO-30 hybrid hydrogels. The SSC assembled with these electrodes pre-immersed in 1 M H_2_SO_4_ for 24 h possesses a *C*_sp_ of 138 F g^−1^ at 0.5 A g^−1^, and an energy density of 20.1 W h kg^−1^ at a power density of 250 W kg^−1^. Biradar *et al.*^130^ prepared 3D PFM-COFs as SSC electrodes using tris(4-formylphenyl) phosphate (P) and 2,6-diaminopyridine (Py), which not only exhibit excellent flame retardancy properties, but also display the highest energy density of 28.44 W h kg^−1^ at 1077.72 W kg^−1^. In summary, the molecular design of COFs is significant for the preparation of multifunctional and efficient SCs. The statistical data are listed in Table 1.

**Table 1:** Device performance summary of SSCs and ASCs

Type	Electrode material	Electrolyte	*C*_sp_ @ current density (F g^−1^ @ A g^−1^)	Energy density (W h kg^−1^)	Power density (W kg^−1^)	Retention (cycles)	Ref.
SSC	TaPa-Py COF/rGO-30	1 M H_2_SO_4_	138 @ 0.5	20.1	250	96% (10 000)	129
				16.8	10 000		
	N-doped C/rGO	6 M KOH	161.8 @ 0.5	14.3	400	86% (3500)	136
	FNC-700	1 M KOH	115 @ 1	31.4	700	100% (10 000)	48
				18.3	7000		
	p-CTF-800	1 M H_2_SO_4_	245.7 @ 0.2	6.91	50	94% (20 000)	127
				5.35	2700		
	SH-COF-1	Et_4_NBF_4_ in MeCN	40 @ 0.5^a^	—	—	—	107
	SH-COF-2		16 @ 0.5^a^	—	—	—	107
	FCTF	1 M H_2_SO_4_	173 @ 1.0	54	14 060	98% (10 000)	126
	TpOMe-DAQ COF	H_2_SO_4_/PVA gel	84 @ 0.25	2.9^b^	61.8^c^	65% (50 000)	89
ASC	Phos-COF-1	3 M KOH	46 @ 1.0	16.6	800	99.95% (28 000)	131
	[C60]0.05-COF	1 M Na_2_SO_4_	47.6 @ 1.0	21.4	900	99% (5000)	137
	rGO/C/MnO_2_	3 M KOH	215.2 @ 0.5	21.2	190.4	72% (2500)	132
	PDC-MA-COF	6 M KOH	335 @ 1.0	29.2	750	88% (2000)	138
	4KT-Tp COF	1 M H_2_SO_4_	583 @ 0.2	12.5	240	92% (20 000)	41
	PANI/TCOF-2	1 M H_2_SO_4_	275 @ 0.5	24.4	200	50% (1000)	59
	N-doped C/g-C_3_N_4_	6 M KOH	129.3 @ 1.0	45.97	659.3	86.5% (2000)	139
				24.7	3703		
	DAAQ-COFs/GA	1 M H_2_SO_4_	112 @ 1.0	30.5	700	88.9% (20 000)	102
				22.8	7000		
	CNT/NKCOF-2	H_2_SO_4_/PVA gel	263 @ 1.0	71	42 000	90% (1000)	44
	Hex-Aza-COF-3	H_2_SO_4_/PVA gel	64 @ 1.0	23.3	661.2	89% (7500)	82
	AAm-TPB	1 M H_2_SO_4_	281@ 1.0	19.16	350	92% (10 000)	93
				11.09	7000		
	Ni-COF	3 M KOH	417 @ 1.0	130	839	94% (10 000)	110
	DBT-MA-COF	6 M KOH	90 @ 1.0	32	800	83% (30 000)	106
	Sb-COF	1 M KOH	78.6 @ 3.0	69	3024	100% (100 000)	113
	TTF-COF1	3 M KOH	183 @ 1.0	57	858	90% (1000)	105
				29	5813		

a Unit of mF cm^−2^ @ mA cm^−2^.

b Unit of μW h cm^−2^.

c Unit of μW cm^−2^.

### 4.2 Asymmetric SCs

Asymmetric supercapacitors (ASCs) compensate for the shortages of EDLCs and PCs by combining two electrodes with different redox reactions or charge storage mechanisms. ASCs not only extend the capacitor's operating voltage window, but also enhance the energy density without decreasing the high power density, thus better meeting the requirements for the power supply system in practical applications. Quinone-based materials, such as benzoquinone or anthraquinone derivatives, can undergo reversible redox reactions during charge/discharge cycles. These redox reactions contribute to PC which allows for higher energy density. COF-incorporating benzoquinone building blocks are applied in ASCs.^105^ Li *et al.* introduced ketone groups into COFs stepwise with increased redox-active sites. The electrodes based on 2KT-Tp COF and 4KT-Tp COF in ASC devices using 1 M aq. H_2_SO_4_ as electrolyte demonstrate significantly increased specific capacitances of 86 F g^−1^ and 251 F g^−1^, respectively, at a current density of 0.8 A g^−1^.^41^ Furthermore, their devices exhibit exceptional stability, with a minimal capacitance decay of approximately 1% after undergoing 10 000 charge/discharge cycles at a current density of 5 A g^−1^. CV curves illustrate the different redox processes of the building blocks, which are composed of 2KT-Tp COF and 4KT-Tp COF. Moreover, by compositing graphene aerogel with anthraquinone-based COFs to form a DAAQ-COFs/GA composite and 1 M aq. H_2_SO_4_ as electrolyte, An *et al.* investigated their electrode performance in ASCs (Fig. 15d). From the CV curves at different scan rates of 5, 10, 20, 30, 50, 70 and 100 mV s^−1^ (Fig. 15e), no obvious aberration is observed in ASCs with the increase of the scan rate, which demonstrates the capability of the fast charging and discharging. Besides, the device can still reach a capacitance retention of 88.9% of its initial value after 20 000 charge/discharge cycles (Fig. 15f). What's more, two ASCs connected in series can successfully light up a “COF” panel consisting of 55 LEDs (Fig. 15f inset). High energy density up to 30.5 W h kg^−1^ is also obtained at a power density of 700 W kg^−1^ (Fig. 15g).^102^

Metal oxides (*e.g.*, RuO_2_, MnO_2_) with abundant surface sites for redox reactions are important as electrode materials for SCs.^37^ Researchers have tried to combine COFs with metal oxides to enhance the performance of ASCs. Sajjad *et al.* constructed SCs using MnSe_2_/rGO//Phos-COF-1 as electrodes and 3 M KOH as electrolyte which achieved a *C*_sp_ of 46·F g^−1^ @ 1 A g^−1^ and remarkable cycling stability (with 99.95% retention after 28 000 cycles).^131^ Zhang *et al.* demonstrated that rGO/C/MnO_2_ as a positive electrode material could deliver a high power density (3.6 kW kg^−1^ at 3.2 A g^−1^) and a high energy density (21.2 W h kg^−1^ at 0.16 A g^−1^) when using LiCl/PVA gel electrolyte.^132^ Kandambeth *et al.* used a RuO_2_/Hex-Aza-COF-3 electrode and H_2_SO_4_/PVA gel electrolyte to assemble high-performance ASCs with a *C*_sp_ of 64 F g^−1^ at 1 A g^−1^, energy density of 23.3 W h kg^−1^ and power density of 661.2 W kg^−1^.^82^

In addition to the mentioned COFs applied in ASCs, various COFs offer potential redox sites for fabricating electrodes. An arylamine-linked COF (AAm-TPB) with abundant electroactive diphenylamine moieties shows a high specific capacitance, energy density and power density of 281 F g^−1^, 19.16 W h kg^−1^ and 350 W kg^−1^ at 1.0 A g^−1^, respectively.^93^ The assembled Sb-COF/rGO shows an excellent energy density of 69 W h kg^−1^.^113^ PANI/TCOF-2, CNT/NKCOF-8, Ni-COF *etc.* with diverse redox-active functional groups exhibited high stability and capacitive performances.^44,59,110^

### 4.3 Flexible SCs

Flexible supercapacitors (FSC) are an important research direction in the context of the increasing demand for wearable electronic devices. Typically, FSCs possess outstanding mechanical flexibility and performance stability under harsh conditions. Conventionally synthesized COFs are difficult to process due to their powder nature and insolubility. Therefore, it is of great necessity to enhance the mechanical properties when utilizing COFs as electrode materials for FSCs.

In COF-based FSC devices, combining COFs with carbon materials as electrode materials is a common approach to improve the mechanical properties. In 2020, Xu *et al.* impregnated COF@OHP complexes on microporous carbon nanotube films (CNTF) to construct composite membranes (CHCMs) to serve as electrode materials for FSC with H_3_PO_4_/PVA gel as electrolyte.^133^ The device has a weight capacitance of 14 F g^−1^. Additionally, the GCD curves had no obvious changes when the device was bent to different angles, suggesting that the capacitance performance is very stable. More importantly, five devices in series can light up a 1.5 V LED. However, the impregnation method does not facilitate the formation of large-scale continuous networks. As opposed to impregnating the complexes directly on CNTF to build the materials, Dong *et al.* prepared the electrode materials by implanting COF materials *in situ* on the carbon skeleton. They synthesized COF/carbon composite foams (DAB/GCF) by homogeneous *in situ* implantation of DAB-COF into porous graphene-coated carbon foams (GCFs) *via* the Schiff base reaction.^53^ The mechanical properties of COF composites with a carbon foam support is significantly improved. The synthesized DAB/GCF could be directly cut into small pieces to act as electrodes for assembling flexible symmetric SCs with 1 M H_2_SO_4_ as electrolyte. The as-fabricated device displays a maximum *C*_sp_ of 129.2 F g^−1^ at 0.5 A g^−1^ with a maximum energy density of 4.47 W h kg^−1^ at a power density of 125 W kg^−1^, and retains 3.05 W h kg^−1^ at 2558 W kg^−1^. In addition, the device has excellent cycling stability with no capacitance drop after 20 000 charge/discharge cycles at 10 A g^−1^. More importantly, after the DAB/GCF composites were subjected to a fatigue compression test at 70% strain for 50 cycles, the assembled flexible SSC maintained 76.7% of the initial capacitance after 20 000 cycles at 10 A g^−1^, indicating that it has good mechanical stability.

**Fig. 16:**
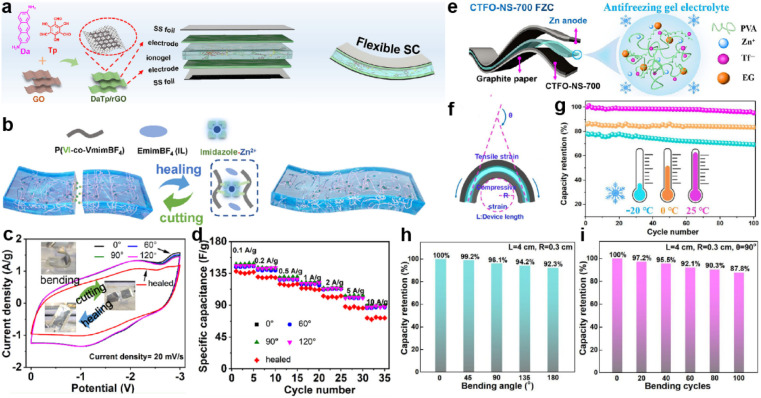
(a) Schematic of FSCs assembled from P_28_/Zn^2+^/2IL ion gel-electrolyte and DaTp/rGO electrodes. (b) Schematic of the spontaneous self-healing ionogel using Im-Zn^2+^ interaction as a dynamic motif. (c) CV curves of FSC with different bending angles and healed FSC. (d) Rate performance of FSCs with different bending angles and the healed sample. Reprinted from ref. 132. Copyright 2023, Elsevier. (e) Schematic structure of CTFO-NS-700 FZC and freeze-resistant PVA/Zn(Tf)_2_/EG gel electrolyte. (f) Schematic diagrams of the structure and bending state of CTFO-NS-700FZC, (h) capacity retention at different bending angles, and (i) capacity retention after 100 bending cycles. (g) Capacity retention of CTFO-NS-700 FZC at 25 °C, 0 °C, and 20 °C. Reprinted from ref. 138. Copyright 2023, Elsevier.

Combining COFs with rGO to prepare composites as electrodes for FSC is another choice. In 2020, Wang *et al.* used a 2D COF as a spacer to prevent stacking of rGO nanosheets, thus improving the electrode performance.^134^ Two types of FSCs are assembled using the best performing rGO/COF-20 as the electrode material. The first one is a two-dimensional thin film supercapacitor with 1 M H_2_SO_4_ as the electrolyte. The device has a specific capacitance of 74 F g^−1^ or 52 F cm^−3^ at 0.1 A g^−1^. The second type is a 1D fiber supercapacitor with H_3_PO_4_/PVA gel as electrolyte. The device has a volumetric energy density of 7.9 mW h cm^−3^ at the power density of 25 mW cm^−3^. Recently, Wu *et al.* synthesized a novel anthracene-based covalent organic framework (DaTp-COF)/rGO hybrid (DaTp/rGO) as an electrode material to assemble FSC (Fig. 16a).^135^ It is worth mentioning that a novel gel electrolyte (P_28_/Zn^2+^/2IL ion gel) was prepared in this system that enables the self-healing of FSCs (Fig. 16b). This kind of ion gel electrolyte has high mechanical strength and self-healing properties. The device delivers good cycling stability, with a specific capacitance retention of about 80% after 10 000 charge–discharge cycles. The ionic gel can tightly adhere to the electrodes and therefore does not come off when the FSC is bent (Fig. 16c). For the self-healing properties of the device, the FSC is cut into two parts which are then placed in contact with each other without external force to heal. After 90 minutes, the device has repaired itself automatically. The *C*_sp_ of the repaired FSC only slightly decreased from 147 to 138 F g^−1^ at a current density of 0.1 A g^−1^, which indicates good self-healing capability of the FSC (Fig. 16d).

In addition to these works, implanting vertical support materials on the surface of carbon fibers to control the direction of COF growth is also effective. He *et al.* used a strong covalent bridge to integrate COF onto the carbon fiber surface (AC-COF).^100^ The DAAQ (2,6-diaminoanthraquinone) coated on the surface of the carbon fibers is employed as the support to control the COF growth direction. Based on this, a flexible all-solid-state SSC with an areal *C*_sp_ of 715 mF cm^−2^ at 0.5 mA cm^−2^ is assembled using PVA/H_2_SO_4_ gel as the electrolyte. A high energy density of 194.64 μW h cm^−2^ can be obtained at a power density of 0.38 mW cm^−2^, and three devices connected in series can easily power a red LED. In summary, the COFs provide an excellent platform for the development of lightweight free-standing flexible electrodes.

Compared with the above composites that combine 2D COFs with conductive carbon, the incorporation of heteroatom-rich guests into COF pores is also a way to enhance the conductivity of 2D COF materials. Haldar *et al.* incorporated polypyrrole (Ppy) into ordered nanochannels of a polyimide COF.^60^ In this composite, the Ppy chains enable electron transport in the COF pore channel, and the interaction of the Ppy unit with anions can effectively broaden the redox voltage window. The assembled solid-state SC utilizing this electrode acquires a *C*_sp_ of 358 mF cm^−2^ at 1 mA cm^−2^ and good cycling stability with a capacitive retention of 82% after 6000 charge/discharge cycles based on PVA/H_2_SO_4_ gel electrolyte. Similar work has been reported by Wang *et al.*, in which a hollow dioxin-based COF-316 microflower with interconnected hollow petals was synthesized.^32^ Then the microflower was homogeneously laminated with polypyrrole (PPy) *via* a self-templating strategy to build a flexible and transparent electrode for a transparent FSC using PVA/H_2_SO_4_ gel electrolyte. The device exhibits an areal *C*_sp_ of 783 μF cm^−2^ at 3 μA cm^−2^ and long-term cycling stability, with a maximum energy density of 0.027 μW h cm^−2^ at a power density of 0.75 μW cm^−2^.

Apart from the above FSCs with ionic gel electrolytes, COF-based electrodes are also applied in aqueous zinc ion FSCs. In 2020, Yu *et al.* synthesized imide-linked PI-COF as an anode for a flexible, dendrite-free, and high-rate all-zinc ion energy storage device.^140^ A high maximum energy density of 66.5 W h kg^−1^ and supercapacitor-level power densities ranging from 133 to 4782 W kg^−1^ are achieved. This COF is also combined with carbon materials to prepare electrodes. In contrast, Liu *et al.* successfully prepared arch-shaped covalent triazine framework (CTF) nanosheets (CTFO-NS-700) by using a bis-nitrile derivative grafting template method with hollow mesoporous SiO_2_ nanospheres (HMSN-CN_2_) as precursors for ZnCl_2_ mediated cyclo-trimerization reaction.^141^ The special morphology and structure of the arch-shaped two-dimensional nanosheets effectively enhance the efficient paths of ion diffusion and migration and provide more active centers. The flexible solid-state zinc-ion SC was assembled using CTFO-NS-700 as the cathode, Zn as the anode, and PVA/Zn(CF_3_SO_3_)_2_/ethylene glycol (PVA/Zn(Tf)_2_/EG) as the membrane electrolyte (Fig. 16e). The device achieves a *C*_sp_ of 245.7 F g^−1^ at 1.0 A g^−1^ and a maximum energy density of 130 W h kg^−1^. In addition, the capacity retention rate of the device under bending (*L* = 2 cm, *R* = 0.15 cm, *θ* = 180°) can reach 87.8% after 100 cycles (Fig. 16f, h and i). More importantly, the flexible device maintains 67.9% of the initial specific capacitance after 100 cycles at 1.0 A g^−1^ at −20 °C, demonstrating its excellent cycling stability and frost resistance (Fig. 16g and Table 2).

**Table 2:** Device performance summary of SSCs and ASCs

Type	Electrode material	Electrolyte	*C*_sp_ @ current density	Energy density	Power density	Retention (cycles)	Ref.
FSC	rGO-COF	H_3_PO_4_/PVA gel	57 F cm^−3^ @ 0.05 A cm^−3^	7.9 mW h cm^−3^	25 mW cm^−3^	—	134
				4.4 mW h cm^−3^	1000 mW cm^−3^		
	PI-COF	2 M ZnSO_4_	208 C g^−1^ @ 1 mA cm^−2^	66.5 W h kg^−1^	133 W kg^−1^	82.5% (2000)	73
				23.9 W h kg^−1^	4781 W kg^−1^		
	COF-316@PPy	H_2_SO_4_/PVA gel	783 μF cm^−2^ @ 3 μA cm^−2^	0.027 μW h cm^−2^	0.75 μW cm^−2^	100% (3400)	32
	DAB/GCF	1 M H_2_SO_4_	129.2 F g^−1^ @ 0.5 A g^−1^	4.47 W h kg^−1^	125 W kg^−1^	100% (20 000)	53
				3.05 W h kg^−1^	2558 W kg^−1^		
	AC-COFs	H_2_SO_4_/PVA gel	715 mF cm^−2^ @ 0.5 mA cm^−2^	196.64 μW h cm^−2^	0.38 mW cm^−2^	87% (20 000)	100
				150.27 μW h cm^−2^	0.55 mW cm^−2^		
	DDP60 CTF	EMIMBF_4_	118.0 F g^−1^	147.5 W h kg^−1^	750 W kg^−1^	97% (10 000)	145
	Ppy@IISERP-COF30	H_2_SO_4_/PVA gel	358 mF cm^−2^ @ 1 mA cm^−2^	50 μW h cm^−2^	561 μW cm^−2^	82% (6000)	60
		Et_4_NBF_4_ in MeCN	275 mF cm^−2^ @ 1 mA cm^−2^	124 μW h cm^−2^	4509 μW cm^−2^	—	60
MSC	g-C_34_N_6_-COF/SWCNTs	LiCl/PVA gel	15.2 mF cm^−2^ @ 10 mV s^−1^	7.3 mW h cm^−3^	0.05 W cm^−3^	93.1% (5000)	68
				2.3 mW h cm^−3^	10.4 W cm^−3^		
	ABA-COF	H_2_SO_4_/PVA gel	927.9 F cm^−3^ @ 10 mV s^−1^	63.2 mW h cm^−3^	3.3 W cm^−3^	83.9% (8000)	123
	COF@rGO	H_2_SO_4_/PVA gel	451.96 F g^−1^	44.22 W h kg^−1^	500 W kg^−1^	94.02% (10 000)	143
				12.22 W h kg^−1^	9998 W kg^−1^		
	PAE-NiNiPcF_8_/EG	H_2_SO_4_/PVA gel	28.1 F cm^−3^ @ 2 mV s^−1^	2.5 μW h cm^−2^	321.34 mW cm^−2^	98.1% (10 000)	112
	COF_TAPB–DHPA_	H_2_SO_4_/PVA gel	723.2 F cm^−3^ @ 10 mV s^−1^	90.7 mW h cm^−3^	2.3 W cm^−3^	97% (5000)	144
	IL-COF_TAPB–DHPA_	H_2_SO_4_/PVA gel	1157.9 F cm^−3^ @ 10 mV s^−1^	139.7 mW h cm^−3^	13.6 W cm^−3^	80% (5000)	144
	Co-COF_TAPB–DHPA_	H_2_SO_4_/PVA gel	1790.1 F cm^−3^ @ 10 mV s^−1^	230.4 mW h cm^−3^	5.9 W cm^−3^	80% (5000)	144
	g-C_30_N_6_-COF/SWCNTs	EMIMBF_4_/PVDF-HFP	44.3 mF cm^−2^ @ 5 mV s^−1^	38.5 mW h cm^−3^	0.3 W cm^−3^	95.2% (5000)	142
				2.0 mW h cm^−3^	14.4 W cm^−3^		
	g-C_48_N_6_-COF/SWCNTs	EMIMBF_4_/PVDF-HFP	41.1 mF cm^−2^ @ 5 mV s^−1^	35.7 mW h cm^−3^	0.3 W cm^−3^	95.1% (5000)	142
				2.3 mW h cm^−3^	16.9 W cm^−3^		

### 4.4 Micro-SCs

The development of micro and nanoelectronics has put forward strong requirements for micro energy storage devices, prompting their comprehensive development in the direction of small size, high energy density, long lifetime and easy integration. Micro-supercapacitors (MSC) possess the advantages of excellent electrochemical performance, controllable electron/ion transport behavior, easy integration, *etc.*, which show a great potential as the energy module of self-powered micro–nano-systems.

**Fig. 17:**
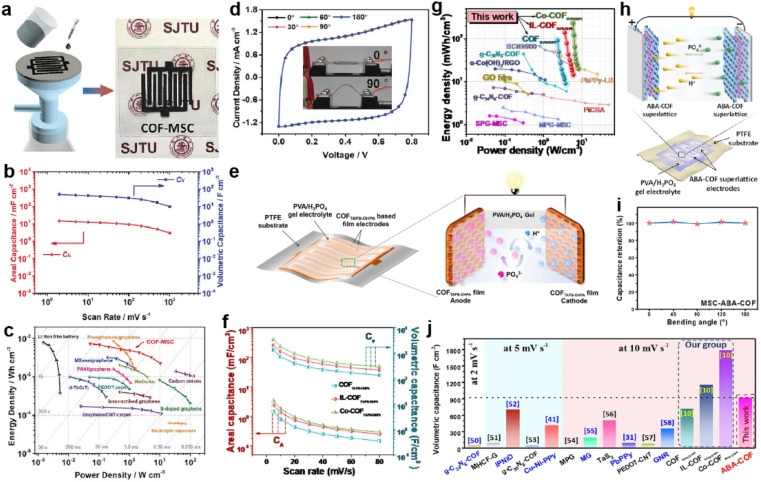
(a) Preparation process and optical photos of a COF-MSC based on g-C_34_N_6_-COF. (b) Specific area capacitance and volume capacitance at different scan rates. (c) Ragone plots compared with some previously reported electrochemical energy storage devices. (d) CV curves under different bending conditions at a scan rate of 50 mV s^−1^ (inset: optical photographs of the COF-MSC under different bending conditions). Reprinted from ref. 68. Copyright 2019, Wiley-VCH Verlag GmbH. (e) Schematic structure of the MSC-COF_TAPB–DHPA_. (f) Specific area capacitance and volume capacitance at different scan rates and (g) Ragone plots with other devices prepared by authors as well as some previously reported devices of MSC-COF_TAPB–DHPA_, MSC-IL-COF_TAPB–DHPA_, and MSC-Co-COF_TAPB–DHPA_. Reprinted from ref. 144. Copyright 2022, Elsevier. (h) Schematic diagram of the in-plane MSC configuration. (i) Capacitance retention under different bending degrees of MSC-ABA-COF. (j) Ragone plots of MSC-ABA-COF, as well as some reported MSC devices. Reprinted from ref. 123. Copyright 2023, Springer Nature, CC BY.

In 2019, Xu *et al.* prepared a novel fully conjugated COF (g-C_34_N_6_-COF) connected *via* unsubstituted C=C bonds, which is combined with carbon nanotubes to act as the electrode of MSCs (Fig. 17a).^68^ Based on the gel LiCl/PVA electrolyte, this device achieves an areal *C*_sp_ of 15.2 mF cm^−2^ at a scan rate of 2 mV s^−1^, and a high energy density of 7.3 mW h cm^−3^ at a power density of 0.05 W cm^−3^, respectively (Fig. 17b and c). More importantly, after 5000 charging/discharging cycles, it still maintains 93.1% of the initial capacitance, indicating excellent cycling stability. Even when the device is bent out of shape, the device still maintains a good CV curve (Fig. 17d). Two more trimethyltriazine-derived olefin-conjugated COFs (g-C_30_N_6_-COF & g-C_48_N_6_-COF) were prepared by the same group in 2020 and composited with SWCNTs to form heterostructured thin films as the electrodes for MSCs (g-C_30_N_6_-COF-MSC & g-C_48_N_6_-COF-MSC).^142^ The areal specific capacitances of the two COF-MSCs are 44.3 mF cm^−2^ and 41.1 mF cm^−2^ at a scan rate of 5 mV s^−1^, and the maximum volumetric energy densities are 38.5 and 35.7 mW h cm^−3^ at 0.3 W cm^−3^, respectively. Additionally, the capacitance retention of the two COF-MSCs were close to 95% after 5000 charge/discharge cycles. Apart from compositing with carbon nanotubes, COFs can also be assembled with rGO or exfoliated graphene (EG) to improve electrochemical performance. Yao *et al.* constructed a flexible MSC using a COF@rGO hybrid film obtained by *in situ* preparation of COF on the surface of 2D rGO nanosheets as an electrode.^143^ An energy density of up to 44.22 W h kg^−1^ and a power density of up to 9998 W kg^−1^ are realized based on PVA/H_2_SO_4_ gel electrolyte. Besides, 94.02% of the initial capacitance remains after 10 000 cycles. Compared with the two previous studies, the carbon nanotubes have a higher aspect ratio and are prone to aggregate, and the conductivity is not as good as that of graphene nanosheets. Therefore, the device assembled with the COF@rGO hybrid film has better performance. Li *et al.* synthesized a series of novel polyarylether-based COFs by direct reaction between various metal-based hexadecafluorophthalocyanines (MPcF_16_, M = Ni, Cu, Zn) and octahydroxyphthalocyanines (PcOH_8_).^112^ Compositing the COFs with exfoliated graphene (EG) can yield self-supporting COF-based thin films with good electrical conductivity. The MSCs are assembled utilizing these films as electrodes and H_2_SO_4_/PVA gel as electrolyte. Among them, PAE-NiNiPcF_8_ MSC provided an ultra-high surface power density of 321.34 mW cm^−2^ and excellent cycling stability.

There have also been some recent studies on the modification of 2D COFs themselves to improve their performance. In 2022, Xu *et al.* fabricated MSCs with Li- and Co-modified imine-linked COF_TAPB–DHPA_.^144^ This COF was synthesized by the Schiff-base reaction of DHPA (2,5-dihydroxyterephthalaldehyde) and TAPB (1,3,5-tris(4-aminophenyl)benzene) while its nanomembranes are prepared by the SMAIS (surfactant-monolayer-assisted interfacial synthesis) method. The modification of cobalt can provide additional pseudocapacitance through the reversible redox reaction of Co(ii/iii). MSCs are assembled using the prepared nanomembranes as electrodes and PVA/H_3_PO_4_ as gel electrolyte (Fig. 17e). As shown in Fig. 17f and g, the MSC assembled with Co-COF_TAPB–DHPA_ films (MSC-Co-COF_TAPB–DHPA_) shows the best performance with an ultra-high volumetric *C*_sp_ of 1790.1 F cm^−3^ at 10 mV s^−1^ and a maximum energy density of 230.4 mW h cm^−3^ (@ 5.9 W cm^−3^). In this work, COFs were modified by metal ion doping, and in the following work, two COFs were alternately assembled to form an “hourglass” steric configuration to improve the material properties. Xu *et al.* prepared mesoporous self-supported COF films with imine bonds (A-COF) and microporous COF films with β-keto-enamine linkage (B-COF) through the SMAIS method.^123^ The ABA-COF superlattices were obtained by assembling them together using layer-by-layer transfer in the presence of van der Waals forces to form a “nano-hourglass” geometry. The A-COF layer acts as an energy storage layer to locally trap the electrolyte and limit the charge release and facilitates the transfer of the electrolyte to the B-COF layer for charge accumulation and depletion at the active site during redox reactions. In addition, two external mesoporous A-COFs protect the microporous B-COFs in case the micropores will not completely withstand the mesoporous highly loaded electrolyte. Therefore, strain of the internal B-COFs and the degradation of the energy storage during the discharge/charge cycling process could be avoided, thus ensuring the energy storage performance of the ABA-COF superlattice.

The optimized ABA-COF superlattice is made into a fork-finger electrode and a flexible MSC device is assembled (MSC-ABA-COF) using H_3_PO_4_/PVA gel as electrolyte (Fig. 17h). The device exhibited a *C*_sp_ of up to 927.9 F cm^−3^ at 10 mV s^−1^ with an energy density of 63.2 mW h cm^−3^. It also exhibits good cycling stability with a capacitance retention of about 83.9% after 8000 charge/discharge cycles. In addition, as shown in Fig. 17i, there is no significant difference in the capacitance values of the device at different bending angles from 0° to 180°, which demonstrates its excellent mechanical flexibility and outstanding stability. The FSC performance is comparable to that of the state-of-the-art devices (Fig. 17j).

## 5. Conclusions and outlook

This year marks the 10th anniversary of COF-based SCs. The rapid development and flourish of both COF materials and devices have been witnessed in the past decade. On the basis of some representative works, the major design strategies of neat COFs and COF composites for performance enhancement are summarized in this review on either a single aspect or overall improvement. The capability of integrating PC and EDLC in a single system guarantees a high theoretical specific capacitance and high energy density. In comparison with the first COF-based SC which obtained a *C*_sp_ of only 48 F g^−1^, a huge improvement has been achieved in specific capacitance which recently reached up to 1875 F g^−1^ in a bulk COF electrode. The combination of a robust skeleton, suitable redox groups, and finely controlled morphology boosts the comprehensive optimization on conductivity, charge and mass transfer, and finally specific capacitance. Meanwhile, the drawbacks of bulk COFs can be solved by combining them with other functional materials. Outstanding rate capability (60% retention from 1 to 500 A g^−1^) and cycling stability (no drop after 10 000 cycles) can be realized in COF composites. Such exciting achievements evidence the great potential of COF-based materials for supercapacitor applications.

Furthermore, the recent advances in device performance and novel device applications are also highlighted. COF-based SCs have high power and energy densities, capacitance retention, and mechanical stability. Besides, functionalized COFs with specific building blocks could be used to enhance the electrochemical performance and stability of SCs. These studies demonstrate that COFs have great potential in developing high-efficiency multifunctional SCs, making them promising materials for future energy storage devices. Nevertheless, some issues in COF-based SCs remain challenging to date.

(1) The relatively low conductivity of COFs is still the main obstacle. Although limited COFs with rational design can reach conductivity in the mS cm^−1^ level, they are still far from that of conductive carbon materials and polymers. The resulting low charge transfer rate and localized charge accumulation will cause a failure in realizing high power density. This also partially induces another problem: (2) trade-off between high energy density and high power density. Besides the former reason, the excessive reference to the design of battery-type COFs to increase the energy density leads to the high ratio of diffusion-controlled redox reactions, thus slowing down the overall capacitive process and expending the power density. (3) Limited theoretical investigation on the energy storage mechanisms. Due to the complicated environment at the interface between the COF-based electrode (especially for COF composites) and electrolyte, it is very difficult to construct a universal model for the quantum simulation. The lack of theoretical guidance also hinders the rational design of COF-based electrode materials. (4) Performance variability. The application of various COF materials in supercapacitors reveals promising electrochemical properties. However, performance variations may occur depending on factors such as the synthesis method, specific COF structure, and electrode fabrication. Ensuring consistent and reproducible performance across different batches of COF-based SCs is essential for practical applications. Efforts to standardize synthesis and fabrication processes can help mitigate this challenge and enhance the reliability of COFs as energy storage materials. (5) Electrolyte. The mainstream electrolytes for SCs (especially MSCs) are inorganic acid or base (such as H_2_SO_4_/PVA), which limit the voltage window of the devices. The utilization of functionalized electrolytes can not only expand the voltage window for improved energy density, but also enable the multifunction of COF-based SCs.

To be honest, the development of COF materials is far behind the device technology. Addressing these potential issues is of utmost importance for the successful integration of COFs into SCs. Researchers are actively engaged in finding solutions to these challenges, aiming to fully unlock the potential of COFs for energy storage applications. The road is tortuous, but the future is bright. As the field of COFs and SCs continues to progress, further research and development hold promise for overcoming these obstacles and maximizing the benefits of COFs in energy storage technologies. The structural and functional improvement of COFs and new energy storage theories will inject new vitality to the design of COF-based materials for SCs.

## Author contributions

All authors contributed to the writing and editing of this review article.

## Conflicts of interest

There are no conflicts to declare.

## Supplementary Material

## References

[cit1] Chu S., Majumdar A. (2012). Nature.

[cit2] Chu S., Cui Y., Liu N. (2017). Nat. Mater..

[cit3] BNEF's Power Transition Trends report, https://assets.bbhub.io/professional/sites/24/BNEF-Power-Transition-Trends-2022_FINAL.pdf, accessed September 2022

[cit4] 2H 2022 Energy Storage Market Outlook, https://about.bnef.com/blog/global-energy-storage-market-to-grow-15-fold-by-2030/, accessed October 2022

[cit5] Choi J. W., Aurbach D. (2016). Nat. Rev. Mater..

[cit6] Wu F. X., Maier J., Yu Y. (2020). Chem. Soc. Rev..

[cit7] Sun J., Xu Y., Lv Y., Zhang Q., Zhou X. (2023). CCS Chem..

[cit8] Wang Y. G., Song Y. F., Xia Y. Y. (2016). Chem. Soc. Rev..

[cit9] Feng Q. K., Zhong S. L., Pei J. Y., Zhao Y., Zhang D. L., Liu D. F., Zhang Y. X., Dang Z. M. (2022). Chem. Rev..

[cit10] Zhou J., Wang B. (2017). Chem. Soc. Rev..

[cit11] Sun J. L., Luo B. C., Li H. X. (2022). Adv. Energy Sustainability Res..

[cit12] Wang F., Wu X., Yuan X., Liu Z., Zhang Y., Fu L., Zhu Y., Zhou Q., Wu Y., Huang W. (2017). Chem. Soc. Rev..

[cit13] Simon P., Gogotsi Y. (2020). Nat. Mater..

[cit14] Zhou Y., Qi H. L., Yang J. Y., Bo Z., Huang F., Islam M. S., Lu X. Y., Dai L. M., Amal R., Wang C. H., Han Z. J. (2021). Energy Environ. Sci..

[cit15] Shao H., Wu Y. C., Lin Z. F., Taberna P. L., Simon P. (2020). Chem. Soc. Rev..

[cit16] Choi C., Ashby D. S., Butts D. M., DeBlock R. H., Wei Q., Lau J., Dunn B. (2020). Nat. Rev. Mater..

[cit17] Yin J., Zhang W. L., Alhebshi N. A., Salah N., Alshareef H. N. (2020). Small Methods.

[cit18] Abdah M., Azman N., Kulandaivalu S., Sulaiman Y. (2020). Mater. Des..

[cit19] Kumar S., Saeed G., Zhu L., Hui K. N., Kim N. H., Lee J. H. (2021). Chem. Eng. J..

[cit20] Geng K. Y., He T., Liu R. Y., Dalapati S., Tan K. T., Li Z. P., Tao S. S., Gong Y. F., Jiang Q. H., Jiang D. L. (2020). Chem. Rev..

[cit21] Kandambeth S., Dey K., Banerjee R. (2019). J. Am. Chem. Soc..

[cit22] Cote A. P., Benin A. I., Ockwig N. W., O'Keeffe M., Matzger A. J., Yaghi O. M. (2005). Science.

[cit23] Wang Z. F., Zhang S. N., Chen Y., Zhang Z. J., Ma S. Q. (2020). Chem. Soc. Rev..

[cit24] Liu X. G., Huang D. L., Lai C., Zeng G. M., Qin L., Wang H., Yi H., Li B. S., Liu S. Y., Zhang M. M., Deng R., Fu Y. K., Li L., Xue W. J., Chen S. (2019). Chem. Soc. Rev..

[cit25] She P., Qin Y., Wang X., Zhang Q. (2022). Adv. Mater..

[cit26] Sun T., Xie J., Guo W., Li D. S., Zhang Q. (2020). Adv. Energy Mater..

[cit27] Keller N., Bein T. (2021). Chem. Soc. Rev..

[cit28] Wang M., Guo H., Xue R., Li Q., Liu H., Wu N., Yao W., Yang W. (2019). ChemElectroChem.

[cit29] DeBlase C. R., Silberstein K. E., Truong T., Abruña H. D., Dichtel W. R. (2013). J. Am. Chem. Soc..

[cit30] Sajjad M., Lu W. (2021). J. Energy Storage.

[cit31] Khayum M A., Vijayakumar V., Karak S., Kandambeth S., Bhadra M., Suresh K., Acharambath N., Kurungot S., Banerjee R. (2018). ACS Appl. Mater. Interfaces.

[cit32] Wang W., Zhao W., Chen T., Bai Y., Xu H., Jiang M., Liu S., Huang W., Zhao Q. (2021). Adv. Funct. Mater..

[cit33] Tao R., Yang T., Wang Y., Zhang J., Wu Z., Qiu L. (2023). Chem. Commun..

[cit34] Wang S., Guo Y., Wang F., Zhou S., Zeng T., Dong Y. (2022). New Carbon Mater..

[cit35] Zhang H., Geng Y., Huang J., Wang Z., Du K., Li H. (2023). Energy Environ. Sci..

[cit36] Xia J., Wang R., Qian C., Sun K., Liu H., Guo C., Li J., Yu F., Bao W. (2022). Crystals.

[cit37] Kandambeth S., Kale V. S., Shekhah O., Alshareef H. N., Eddaoudi M. (2022). Adv. Energy Mater..

[cit38] Jin S., Allam O., Jang S. S., Lee S. W. (2022). InfoMat.

[cit39] Wei S., Wang J., Li Y., Fang Z., Wang L., Xu Y. (2023). Nano Res..

[cit40] Kim J., Kim Y., Yoo J., Kwon G., Ko Y., Kang K. (2023). Nat. Rev. Mater..

[cit41] Li M., Liu J., Li Y., Xing G., Yu X., Peng C., Chen L. (2021). CCS Chem..

[cit42] Khattak A. M., Ghazi Z. A., Liang B., Khan N. A., Iqbal A., Li L., Tang Z. (2016). J. Mater. Chem. A.

[cit43] Bhanja P., Bhunia K., Das S. K., Pradhan D., Kimura R., Hijikata Y., Irle S., Bhaumik A. (2017). ChemSusChem.

[cit44] Yang Y., Zhang P., Hao L., Cheng P., Chen Y., Zhang Z. (2021). Angew. Chem., Int. Ed..

[cit45] Patra B. C., Bhattacharya S. (2021). Chem. Mater..

[cit46] Xu F., Xu H., Chen X., Wu D., Wu Y., Liu H., Gu C., Fu R., Jiang D. (2015). Angew. Chem., Int. Ed..

[cit47] Liu Y., Zhou W. Q., Teo W. L., Wang K., Zhang L. Y., Zeng Y. F., Zhao Y. L. (2020). Chem.

[cit48] Martín Illán J. Á., Sierra L., Ocón P., Zamora F. (2022). Angew. Chem., Int. Ed..

[cit49] Wang P., Wu Q., Han L., Wang S., Fang S., Zhang Z., Sun S. (2015). RSC Adv..

[cit50] Sun B., Liu J., Cao A., Song W., Wang D. (2017). Chem. Commun..

[cit51] Han Y., Zhang Q., Hu N., Zhang X., Mai Y., Liu J., Hua X., Wei H. (2017). Chin. Chem. Lett..

[cit52] An N., Guo Z., Guo C., Wei M., Sun D., He Y., Li W., Zhou L., Hu Z., Dong X. (2023). Chem. Eng. J..

[cit53] Dong Y., Wang Y., Zhang X., Lai Q., Yang Y. (2022). Chem. Eng. J..

[cit54] Mulzer C. R., Shen L., Bisbey R. P., McKone J. R., Zhang N., Abruña H. D., Dichtel W. R. (2016). ACS Cent. Sci..

[cit55] Wu Y., Yan D., Zhang Z., Matsushita M. M., Awaga K. (2019). ACS Appl. Mater. Interfaces.

[cit56] Snook G. A., Kao P., Best A. S. (2011). J. Power Sources.

[cit57] Liu S., Yao L., Lu Y., Hua X., Liu J., Yang Z., Wei H., Mai Y. (2019). Mater. Lett..

[cit58] Peng C., Yang H., Chen S., Wang L. (2020). J. Energy Storage.

[cit59] Dutta T. K., Patra A. (2021). Chem.–Asian J..

[cit60] Haldar S., Rase D., Shekhar P., Jain C., Vinod C. P., Zhang E., Shupletsov L., Kaskel S., Vaidhyanathan R. (2022). Adv. Energy Mater..

[cit61] Peng Y. W., Zhao M. T., Chen B., Zhang Z. C., Huang Y., Dai F. N., Lai Z. C., Cui X. Y., Tan C. L., Zhang H. (2018). Adv. Mater..

[cit62] Peng H., Raya J., Richard F., Baaziz W., Ersen O., Ciesielski A., Samorì P. (2020). Angew. Chem., Int. Ed..

[cit63] Peng H., Huang S., Tranca D., Richard F., Baaziz W., Zhuang X., Samorì P., Ciesielski A. (2021). ACS Nano.

[cit64] Huang N., Wang P., Jiang D. (2016). Nat. Rev. Mater..

[cit65] Diercks C. S., Yaghi O. M. (2017). Science.

[cit66] Kandambeth S., Mallick A., Lukose B., Mane M. V., Heine T., Banerjee R. (2012). J. Am. Chem. Soc..

[cit67] Zhuang X., Zhao W., Zhang F., Cao Y., Liu F., Bi S., Feng X. (2016). Polym. Chem..

[cit68] Xu J., He Y., Bi S., Wang M., Yang P., Wu D., Wang J., Zhang F. (2019). Angew. Chem., Int. Ed..

[cit69] Pyles D. A., Crowe J. W., Baldwin L. A., McGrier P. L. (2016). ACS Macro Lett..

[cit70] Waller P. J., AlFaraj Y. S., Diercks C. S., Jarenwattananon N. N., Yaghi O. M. (2018). J. Am. Chem. Soc..

[cit71] Li T., Yan X., Liu Y., Zhang W., Fu Q., Zhu H., Li Z., Gu Z. (2019). Polym. Chem..

[cit72] El Mahdy A. F. M., Hung Y. H., Mansoure T. H., Yu H. H., Chen T., Kuo S. W. (2019). Chem.–Asian J..

[cit73] Yu M. H., Chandrasekhar N., Raghupathy R., Ly K. H., Zhang H. Z., Dmitrieva E., Liang C. L., Lu X. H., Kuhne T. D., Mirhosseini H., Weidinger I. M., Feng X. L. (2020). J. Am. Chem. Soc..

[cit74] Yao L. Y., Ma C., Sun L. B., Zhang D. L., Chen Y. Z., Jin E. Q., Song X. W., Liang Z. Q., Wang K. X. (2022). J. Am. Chem. Soc..

[cit75] Kim T., Lee J., Kim N., Lee S., Gu M., Kim B. (2022). Chem. Commun..

[cit76] Guan X., Li H., Ma Y., Xue M., Fang Q., Yan Y., Valtchev V., Qiu S. (2019). Nat. Chem..

[cit77] Zhang B., Wei M., Mao H., Pei X., Alshmimri S. A., Reimer J. A., Yaghi O. M. (2018). J. Am. Chem. Soc..

[cit78] Yang C., Jiang K., Zheng Q., Li X., Mao H., Zhong W., Chen C., Sun B., Zheng H., Zhuang X., Reimer J. A., Liu Y., Zhang J. (2021). J. Am. Chem. Soc..

[cit79] Meng Z., Aykanat A., Mirica K. A. (2019). Chem. Mater..

[cit80] Li X., Wang H., Chen H., Zheng Q., Zhang Q., Mao H., Liu Y., Cai S., Sun B., Dun C., Gordon M. P., Zheng H., Reimer J. A., Urban J. J., Ciston J., Tan T., Chan E. M., Zhang J., Liu Y. (2020). Chem.

[cit81] Shehab M. K., Weeraratne K. S., Huang T., Lao K. U., El-Kaderi H. M. (2021). ACS Appl. Mater. Interfaces.

[cit82] Kandambeth S., Jia J., Wu H., Kale V. S., Parvatkar P. T., Czaban Jóźwiak J., Zhou S., Xu X., Ameur Z. O., Abou Hamad E., Emwas A. H., Shekhah O., Alshareef H. N., Eddaoudi M. (2020). Adv. Energy Mater..

[cit83] Iqbal R., Majeed M. K., Hussain A., Ahmad A., Ahmad M., Jabar B., Akbar A. R., Ali S., Rauf S., Saleem A. (2023). Mater. Chem. Front..

[cit84] Arunan E., Desiraju G. R., Klein R. A., Sadlej J., Scheiner S., Alkorta I., Clary D. C., Crabtree R. H., Dannenberg J. J., Hobza P., Kjaergaard H. G., Legon A. C., Mennucci B., Nesbitt D. J. (2011). Pure Appl. Chem..

[cit85] Chandra S., Roy Chowdhury D., Addicoat M., Heine T., Paul A., Banerjee R. (2017). Chem. Mater..

[cit86] Qian C., Feng L. L., Teo W. L., Liu J. W., Zhou W., Wang D. D., Zhao Y. L. (2022). Nat. Rev. Chem.

[cit87] Xu H., Gao J., Jiang D. (2015). Nat. Chem..

[cit88] Halder A., Karak S., Addicoat M., Bera S., Chakraborty A., Kunjattu S. H., Pachfule P., Heine T., Banerjee R. (2018). Angew. Chem., Int. Ed..

[cit89] Halder A., Ghosh M., Khayum M A., Bera S., Addicoat M., Sasmal H. S., Karak S., Kurungot S., Banerjee R. (2018). J. Am. Chem. Soc..

[cit90] Haldar S., Kushwaha R., Maity R., Vaidhyanathan R. (2019). ACS Mater. Lett..

[cit91] Lin Y. Z., Fan H. J., Li Y. F., Zhan X. W. (2012). Adv. Mater..

[cit92] Ando S., Murakami R., Nishida J., Tada H., Inoue Y., Tokito S., Yamashita Y. (2005). J. Am. Chem. Soc..

[cit93] Yang Z., Liu J., Li Y., Zhang G., Xing G., Chen L. (2021). Angew. Chem., Int. Ed..

[cit94] Liu Y., Li X., Wang S., Cheng T., Yang H., Liu C., Gong Y., Lai W., Huang W. (2020). Nat. Commun..

[cit95] Zhang Y., Zhang B., Chen L., Wang T., Di M., Jiang F., Xu X., Qiao S. (2022). J. Colloid Interface Sci..

[cit96] Wei H., Ning J., Cao X., Li X., Hao L. (2018). J. Am. Chem. Soc..

[cit97] DeBlase C. R., Hernández-Burgos K., Silberstein K. E., Rodríguez-Calero G. G., Bisbey R. P., Abruña H. D., Dichtel W. R. (2015). ACS Nano.

[cit98] Zha Z., Xu L., Wang Z., Li X., Pan Q., Hu P., Lei S. (2015). ACS Appl. Mater. Interfaces.

[cit99] Sun J., Klechikov A., Moise C., Prodana M., Enachescu M., Talyzin A. V. (2018). Angew. Chem., Int. Ed..

[cit100] He Y., An N., Meng C., Xiao L., Wei Q., Zhou Y., Yang Y., Li Z., Hu Z. (2022). ACS Appl. Mater. Interfaces.

[cit101] Li C., Yang J., Pachfule P., Li S., Ye M., Schmidt J., Thomas A. (2020). Nat. Commun..

[cit102] An N., Guo Z., Xin J., He Y., Xie K., Sun D., Dong X., Hu Z. (2021). J. Mater. Chem. A.

[cit103] Wu Z., Adekoya D., Huang X., Kiefel M. J., Xie J., Xu W., Zhang Q., Zhu D., Zhang S. (2020). ACS Nano.

[cit104] Segura J. L., Martín N. (2001). Angew. Chem., Int. Ed..

[cit105] Li T., Yan X., Zhang W., Han W., Liu Y., Li Y., Zhu H., Li Z., Gu Z. (2020). Chem. Commun..

[cit106] Li L., Lu F., Guo H., Yang W. (2021). Microporous Mesoporous Mater..

[cit107] Pakulski D., Montes-García V., Gorczyński A., Czepa W., Chudziak T., Samorì P., Ciesielski A. (2022). J. Mater. Chem. A.

[cit108] Sajjad M., Tao R., Qiu L. (2021). J. Mater. Sci.: Mater. Electron..

[cit109] Lee D. Y., Yoon S. J., Shrestha N. K., Lee S., Ahn H., Han S. (2012). Microporous Mesoporous Mater..

[cit110] Li T., Zhang W., Liu Y., Li Y., Cheng C., Zhu H., Yan X., Li Z., Gu Z. (2019). J. Mater. Chem. A.

[cit111] Yusran Y., Li H., Guan X., Li D., Tang L., Xue M., Zhuang Z., Yan Y., Valtchev V., Qiu S., Fang Q. (2020). Adv. Mater..

[cit112] Li N., Jiang K., Rodríguez Hernández F., Mao H., Han S., Fu X., Zhang J., Yang C., Ke C., Zhuang X. (2022). Adv. Sci..

[cit113] Kang K., Wu Z., Zhao M., Li Z., Ma Y., Zhang J., Wang Y., Sajjad M., Tao R., Qiu L. (2022). Chem. Commun..

[cit114] Mullangi D., Shalini S., Nandi S., Choksi B., Vaidhyanathan R. (2017). J. Mater. Chem. A.

[cit115] Shen J., Jiang Q. Y., Zhong G. Q. (2007). Prog. Chem..

[cit116] Tao R., Shen X., Hu Y., Kang K., Zheng Y., Luo S., Yang S., Li W., Lu S., Jin Y., Qiu L., Zhang W. (2020). Small.

[cit117] Wang G., Zhang L., Zhang J. (2012). Chem. Soc. Rev..

[cit118] Wang Z., Li H., Tang Z., Liu Z., Ruan Z., Ma L., Yang Q., Wang D., Zhi C. (2018). Adv. Funct. Mater..

[cit119] Bhat T. S., Patil P. S., Rakhi R. B. (2022). J. Energy Storage.

[cit120] Jayaramulu K., Dubal D. P., Nagar B., Ranc V., Tomanec O., Petr M., Datta K. K. R., Zboril R., Gómez-Romero P., Fischer R. A. (2018). Adv. Mater..

[cit121] Kushwaha R., Haldar S., Shekhar P., Krishnan A., Saha J., Hui P., Vinod C. P., Subramaniam C., Vaidhyanathan R. (2021). Adv. Energy Mater..

[cit122] Chatterjee A., Sun J., Rawat K. S., Van Speybroeck V., Van Der Voort P. (2023). Small.

[cit123] Xu X., Zhang Z., Xiong R., Lu G., Zhang J., Ning W., Hu S., Feng Q., Qiao S. (2023). Nano-Micro Lett..

[cit124] Xu Q., Tang Y., Zhai L., Chen Q., Jiang D. (2017). Chem. Commun..

[cit125] Wu C., Zhang H., Hu M., Shan G., Gao J., Liu J., Zhou X., Yang J. (2020). Adv. Electron. Mater..

[cit126] Gao Y., Zhi C., Cui P., Zhang K. A. I., Lv L., Wang Y. (2020). Chem. Eng. J..

[cit127] Gao Y., Cui P., Liu J., Sun W., Chen S., Chou S., Lv L., Wang Y. (2021). ACS Appl. Energy Mater..

[cit128] Ma L., Zhang W., Zhang R., Niu H., Yang Q., Li F., Zhou M., Zhang L., Huang Y. (2023). Appl. Surf. Sci..

[cit129] Wang C., Liu F., Yan S., Liu C., Yu Z., Chen J., Lyu R., Wang Z., Xu M., Dai S., Chen Y., Wei L. (2022). Carbon.

[cit130] Biradar M. R., Rao C. R. K., Bhosale S. V., Bhosale S. V. (2023). Energy Fuels.

[cit131] Sajjad M., Ismail J., Shah A., Mahmood A., Shah M. Z. U., Rahman S. U., Lu W. (2021). J. Energy Storage.

[cit132] Zhang H., Lin L., Wu B., Hu N. (2020). J. Power Sources.

[cit133] Xu Z., Liu Y., Wu Z., Wang R., Wang Q., Li T., Zhang J., Cheng J., Yang Z., Chen S., Miao M., Zhang D. (2020). Chem. Eng. J..

[cit134] Wang C., Liu F., Chen J., Yuan Z., Liu C., Zhang X., Xu M., Wei L., Chen Y. (2020). Energy Storage Mater..

[cit135] Wu J., Huang L., Wang S., Li X., Wen L., Li X., Feng T., Li P., Fang Z., Wu M., Lv W. (2023). Energy Storage Mater..

[cit136] Ibrahim M., Abdelhamid H. N., Abuelftooh A. M., Mohamed S. G., Wen Z., Sun X. (2022). J. Energy Storage.

[cit137] Zhao X., Sajjad M., Zheng Y., Zhao M., Li Z., Wu Z., Kang K., Qiu L. (2021). Carbon.

[cit138] Li L., Lu F., Xue R., Ma B., Li Q., Wu N., Liu H., Yao W., Guo H., Yang W. (2019). ACS Appl. Mater. Interfaces.

[cit139] Ibrahim M., Fayed M. G., Mohamed S. G., Wen Z., Sun X., Abdelhamid H. N. (2022). ACS Appl. Energy Mater..

[cit140] Yu M., Chandrasekhar N., Raghupathy R. K. M., Ly K. H., Zhang H., Dmitrieva E., Liang C., Lu X., Kühne T. D., Mirhosseini H., Weidinger I. M., Feng X. (2020). J. Am. Chem. Soc..

[cit141] Liu B., Quan T., Yang M., Liu Y., Chen H., Li H. (2023). Chem. Eng. J..

[cit142] Zhang F., Wei S., Wei W., Zou J., Gu G., Wu D., Bi S., Zhang F. (2020). Sci. Bull..

[cit143] Yao M., Guo C., Geng Q., Zhang Y., Zhao X., Zhao X., Wang Y. (2022). Ind. Eng. Chem. Res..

[cit144] Xu X., Xiong R., Zhang Z., Zhang X., Gu C., Xu Z., Qiao S. (2022). Chem. Eng. J..

[cit145] Mahato M., Nam S., Tabassian R., Oh S., Nguyen V. H., Oh I. K. (2022). Adv. Funct. Mater..

